# A test of local adaptation to drought in germination and seedling traits in populations of two alpine forbs across a 2000 mm/year precipitation gradient

**DOI:** 10.1002/ece3.9772

**Published:** 2023-02-07

**Authors:** Ragnhild Gya, Sonya Rita Geange, Joshua Scott Lynn, Joachim Paul Töpper, Øystein Wallevik, Camilla Zernichow, Vigdis Vandvik

**Affiliations:** ^1^ Department of Biological Sciences University of Bergen Bergen Norway; ^2^ Bjerknes Center for Climate Research Bergen Norway; ^3^ Norwegian Institute for Nature Research Bergen Norway

**Keywords:** biomass allocation, polyethylene glycol, seed mass, time to 50% germination, time to max germination

## Abstract

Seed regeneration is a critical stage in the life histories of plants, affecting species' abilities to maintain local populations, evolve, and disperse to new sites. In this study, we test for local adaptations to drought in germination and seedling growth of two alpine forbs with contrasting habitat preferences: the alpine generalist *Veronica alpina* and the snowbed specialist *Sibbaldia procumbens*. We sampled seeds of each species from four populations spanning a precipitation gradient from 1200 to 3400 mm/year in western Norway. In a growth chamber experiment, we germinated seeds from each population at 10 different water potentials under controlled light and temperature conditions. Drought led to lower germination percentage in both species, and additionally, slower germination, and more investment in roots for *V. alpina.* These responses varied along the precipitation gradient. Seeds from the driest populations had higher germination percentage, shorter time to germination, and higher investments in the roots under drought conditions than the seeds from the wettest populations – suggesting local adaption to drought. The snowbed specialist, *S. procumbens*, had lower germination percentages under drought, but otherwise did not respond to drought in ways that indicate physiological or morphological adaptions to drought. *S. procumbens* germination also did not vary systematically with precipitation of the source site, but heavier‐seeded populations germinated to higher rates and tolerated drought better. Our study is the first to test drought effects on seed regeneration in alpine plants populations from high‐precipitation regions. We found evidence that germination and seedling traits may show adaptation to drought even in populations from wet habitats. Our results also indicate that alpine generalists might be more adapted to drought and show more local adaptations in drought responses than snowbed specialists.

## 1 INTRODUCTION

Species are generally expected to respond to changes in the abiotic and biotic environments in line with their niche requirements (Colwell & Rangel, 2009; Davis, 1969; Hutchinson, 1957; Whittaker, 1956). The majority of the empirical tests of niche theory focus on adults, ignoring the important seed regeneration stage (Briceño et al., 2015; Copenhaver‐Parry et al., 2017; Grime & Hillier, 2009; Larson & Funk, 2016). The existing regeneration stage literature documents niche‐related variation among plant species in their germination responses to abiotic gradients like temperature (Canham & Murphy, 2016; Fernández‐Pascual et al., 2021; Rehm et al., 2015; Vandvik et al., 2017) and moisture (Albrecht & McCarthy, 2009; Ibáñez et al., 2007). However, intraspecific variation in germination responses to abiotic gradients has received less attention.

Populations within species may vary in their germination responses along environmental variables as a consequence of local adaptations (Grassein et al., 2014; Kawecki & Ebert, 2004), for instance, by optimizing germination timing and rate to population‐specific environmental conditions (Baskin & Baskin, 2014; Giménez‐Benavides et al., 2007; Meineri et al., 2013; Satyanti et al., 2019). The selective pressures operating on such local adaptations may generally be expected to be high in alpine ecosystems due to harsh environmental conditions, topographically complex landscapes, and high microclimate variability (Scherrer & Körner, 2011). This environmental variability within the alpine habitats leads to high but spatiotemporally variable seedling mortality rates (Graae et al., 2018; Scherrer & Körner, 2011), which further suggests selection for locally adapted seed regeneration responses in alpine species (Giménez‐Benavides et al., 2007; Kim & Donohue, 2013; Mondoni et al., 2009). While such local adaptions may increase the total range of conditions under which the species as a whole can survive, each locally adapted population tolerates a narrower range of conditions and can be vulnerable to climate change, especially if dispersal is low (Atkins & Travis, 2010; Valladares et al., 2014). Local adaptations could therefore confer a higher vulnerability to climate change of each population than inferred from the species‐wide geographic distribution and climatic range (Atkins & Travis, 2010; Diamond & Martin, 2020; Peterson et al., 2018, 2019).

Studies of climate change impacts on ecosystems generally tend to focus on changes in mean conditions of single climatic factors (Wu et al., 2011). However, climate extremes (Román‐Palacios & Wiens, 2020), microclimates (Graae et al., 2011), or variations in the timing of climatic events (Hülber et al., 2011) may be more important than changes in climatic means for individual's fitness, especially during the vulnerable seed regeneration stage. Climate change studies on ecosystems and biodiversity often focus on specific habitats for certain climatic factors. For example, drought effects on germination are more commonly studied in water‐limited systems (e.g., Adams, 1999; Cochrane et al., 2014). However, studies on germination in temperature‐limited arctic and alpine systems often focus on the impacts of warming (e.g., Fernández‐Pascual et al., 2021; Hoyle et al., 2013; Mondoni et al., 2012). Less common, though still critical, are studies examining drought responses in landscapes not traditionally subject to water limitation that might experience more frequent droughts as climate change progresses.

The rate and even direction of precipitation changes are regionally variable, and for oceanic boreal regions like Norway, we are expecting a general increase in precipitation (Hanssen‐Bauer et al., 2017). In alpine regions, much of the increased precipitation falls as snow. But as increased temperature leads to earlier snow melt‐out, increased evapotranspiration, and increased run‐off, this can paradoxically result in higher risks of early summer soil moisture deficit in the alpine in the future (Hanssen‐Bauer et al., 2017). This is also the period when seed germination and seedling establishment occur in alpine ecosystems (Mondoni et al., 2012). Thus, the question emerges: how will plant recruitment in high‐precipitation alpine systems respond to increased incidence and severity of drought conditions during the critical recruitment phase and what role may local adaptations play in these responses?

Plant functional trait‐based studies increasingly provide insights into niche theory (Treurnicht et al., 2019), local adaptations (Gonzalo‐Turpin & Hazard, 2009; Joshi et al., 2001), and more generally, link plant performance to abiotic factors like drought (i.e., Jung et al., 2014). For example, plants from drier habitats tend to produce larger seeds in order to provide more resources for handling drought stress during germination (Buckley, 1982; Leishman & Westoby, 1994; Moles & Westoby, 2004; Wellstein et al., 2013; Wright & Westoby, 1999). Reduced water availability can result in delayed germination (Cochrane et al., 2015; Vázquez‐Ramírez & Venn, 2021) or reduced germination rates (Baskin & Baskin, 2014; Forbis, 2003; Vázquez‐Ramírez & Venn, 2021; Walck et al., 2011), which could be indicative of strategies to postpone germination to a time with more favorable moisture conditions. During seedling establishment, drought might select for resource‐conservative growth strategies including tougher, less resource‐acquisitive leaves, roots, and shoot tissues (Larson et al., 2020), and greater biomass allocation toward root tissue (Freschet et al., 2018; Larson et al., 2020; Larson & Funk, 2016). At the other end of the spectrum, excess water may also act as a stressor during germination and seedling establishment via effects such as reduced temperature, reduced nutrient availability, and increased risk of exposure to anoxic conditions in the soil (Meineri et al., 2013; Tingstad et al., 2015).

To explore local adaptations to drought in the regeneration of alpine species, we conducted a growth chamber experiment to investigate population‐level variation in germination and seedling growth responses to variations in moisture availability. We focused on two alpine species with contrasting habitat selectivity; the alpine generalist *Veronica alpina* and the snow‐bed specialist *Sibbaldia procumbens* (Lid & Lid, 2007; Mossberg & Stenberg, 2012). For each of these species, we collected seeds from four local populations in alpine grasslands in Western Norway, spanning a precipitation gradient from 1200 to 3400 mm/year and an average of 28%–53% soil moisture in the growing season. Based on the arguments outlined above, sampling populations from along a precipitation gradient of two alpine species occupying different niches, we ask the following questions:

1. Do populations of alpine species from climates and microhabitats reflecting different moisture regimes vary in their seed germination response to moisture availability?
2. Are these patterns and responses also reflected in seedling growth and development traits?
3. Are germination and growth responses to moisture availability dependent on seed mass?

We expect higher drought tolerance in populations from drier environments, expressed as different germination and seedling traits. We expect the alpine generalist species to have a higher variation within and between populations than the snowbed specialist because it is found across a larger range of microclimates within each population and thus more exposed to the climatic variability between sites. Additionally, we are interested in how seed size variation across populations may explain drought tolerance responses. The precise wording of the predictions and how we tested them are outlined in the Analysis part of the methods.

## 2 METHODS

### 2.1 Study system and species

**FIGURE 1:**
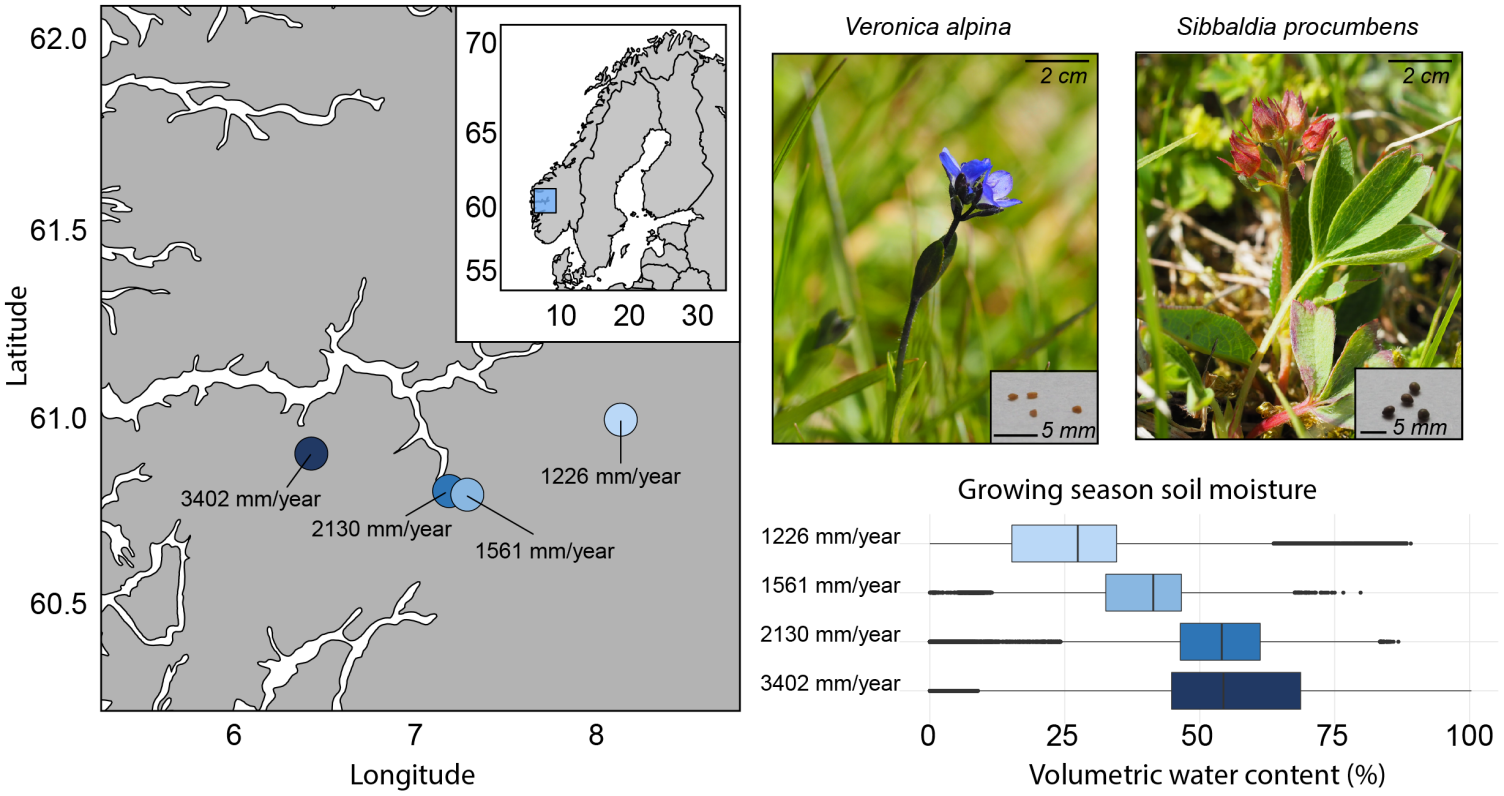
Geographical and climatic information about the location for seed collection of the two study species *Veronica alpina* and *Sibbaldia procumbens*. The sites are situated in southwestern Norway ranging from the wet coastal site with annual mean precipitation of 3402 mm/year to the drier inland site with 1226 mm/year. Precipitation is calculated based on daily means from 2009 to 2019 with data from the Norwegian Meteorological Institute (met.no). Soil moisture at the sites is obtained from the permanent loggers at the sites during the growing season from 2009 to 2019. Photos: Ragnhild Gya.

The sites are semi‐natural grasslands selected to represent a precipitation gradient from the continental, dry inland to the oceanic, humid coast. The precipitation ranges from 1200 to 3400 mm/year while having similar average growing season temperatures (mean temperature of the four warmest months per year of around 7°C) (Figure 1). The precipitation and temperature data are based on daily averages for the period from 2009 to 2019 provided by the Norwegian Meteorological Institute (met.no). We confirmed the relevance of the broad‐scale climate gradient for the microclimatic conditions experienced by the seeds using soil moisture data from permanent local data loggers at the sites, with two sensors about 10 cm in the ground, reading data every hour. The average soil moisture ranged from 28% to 53% (Figure 1) along the gradient measured in the growing season (June–September) from 2009 to 2019. The sites are part of a larger group of climate change experiments and sites were chosen to reflect broad‐scale climate gradients, while keeping all other underlying factors similar (i.e., slope, aspect, soil pH, soil type, vegetation type (semi‐natural grasslands), and grazing history). For more information about the sites, see Klanderud et al. (2015).

We selected the perennial forbs *Veronica alpina* (Plantaginaceae) and *Sibbaldia procumbens* (Rosaceae) as study species because they are common and relatively abundant species across all four sites. *Veronica alpina* is a circumpolar alpine/arctic species (Albach et al., 2006), an alpine generalist growing in a range of habitats from snowbeds to moist and dry alpine grasslands and heaths (Lid & Lid, 2007; Mossberg & Stenberg, 2012). The seeds of *V. alpina* are non‐dormant (Baskin & Baskin, 2014) but show higher germination rates after cold stratification (Bernareggi et al., 2016; Vandvik et al., 2017). *Sibbaldia procumbens* is a also circumpolar alpine/arctic species (Allen et al., 2015) mostly found in snowbeds (Coker, 1966; Lid & Lid, 2007; Mossberg & Stenberg, 2012). *Sibbaldia procumbens* seeds show physiological dormancy with increased germination percentages after cold stratification (Baskin & Baskin, 2014).

For each species and population, we collected mature seeds in August and September 2019. The maturity was assessed by the color of the capsule (*V. alpina*: orange, *S. procumbens*: brown) and the capsule being open or easy to open, following protocol 4.2 Seed Viability, Germinability, and Dormancy in Halbritter et al. (2020).

### 2.2 Seed preparation and cold stratification

After collection, the seed material was cleaned of debris and stored dry at room temperature (20°C) for 3 months. Seeds were then cold stratified to break dormancy (Shimono & Kudo, 2005) for 9 weeks at 4°C. They were kept moist using wet filter paper in Petri dishes sealed with parafilm, which were individually wrapped in aluminum foil to exclude light.

### 2.3 Germination experiment

To test how germination is affected by water availability, we germinated the seeds across 10 different water potentials in the range between −0.25 (only agar) and −1.7 MPa. The range was chosen based on previous literature showing that −1.7 MPa is past or close to many species' threshold for germination and survival (i.e., Cochrane et al., 2014; Evans & Etherington, 1990; Hovenden et al., 2008; Van Der Weele et al., 2000). To our knowledge, there are no drought germination studies on our study species (*V. alpina* and *S. procumbens*); therefore, by testing across such a large gradient, we aimed to identify the species' minimum water potential for germination. We used 1% agar which was sterilized in an autoclave before the experiment to minimize fungal and bacterial growth during the germination trial. By using agar, we were able to keep a constant and highly controlled water potential over time, avoiding problems with drying out when using filter paper, or fluctuating moisture when using soils (Osmolovskaya et al., 2018). We used polyethylene glycol (PEG, molecular weight 8000; Sigma) to alter the osmotic water potential, with PEG introduced to the agar media via diffusion (following the protocol from Van Der Weele et al., 2000). We poured approximately 30 mL of PEG solution on top of 20 mL volume of agar. After 4 days, the solution diffused into the agar and the remaining solution was poured off. To be able to assess seedlings' germination and development based on the maternal resources invested in the seeds from plants growing in their home climates, we did not add nutrients to the agar.

The germination experiment was conducted in two growth chambers (Sanyo Incubator MIR‐553) due to space limitations. The chambers had a fluctuating day/night temperature regime of 25°C/10°C at 16/8 h, with 25°C and light in the 16‐h day, and 10°C and darkness in the 8‐h night. The long day and short nights simulate the light conditions of the Norwegian summer, and the temperatures are used to ensure good germination as several previous studies from alpine systems in the region find optimal germination rates and percentages at fluctuating light and temperatures of 20–25°C (Graae et al., 2008; Vandvik et al., 2017). To account for the potential within‐ and between‐chamber variation in temperature and light environment, we systematically rotated the Petri dishes two times per week within and between the two growth chambers.

For each species and population combination, we sowed 20 seeds in nine replicate Petri dishes for each water potential treatment (2 species × 4 populations × 10 drought treatments × 9 replicates × 20 seeds). For *S. procumbens* at the second wettest site, limited seed collection only allowed for 10 seeds to be used per Petri dish and only seven water potential treatments, with nine replicates per treatment.

We scored germination daily from the onset because of the rapid germination rate expected in *V. alpina* based on previous literature (Bernareggi et al., 2016; Vandvik et al., 2017), but reduced scoring effort to two times a week once the cumulative germination curves started flattening. The seeds were scored as germinated once radicle protrusion and elongation were visible to the naked eye. We also scored the date for cotyledon and the first true leaf emergence. These were scored once the cotyledons or true leaves were fully expanded. Germinants continued growing in the Petri dish until the first true leaf was fully expanded (*S. procumbens*) or until 1 week after the first pair of true leaves have fully expanded (*V. alpina*). The differences in the protocol for the two species are due to the small size of the leaves of *V. alpina* when they first emerge. We stopped the experiment when 20 weeks had passed and harvested all the remaining seedlings and obtained above‐ and belowground biomass, even if they did not reach the true leaf stage.

At the end of the experiment, we assessed the viability of seeds that had still not germinated by using an embryo integrity (squish) test and/or the cut test. We assumed that the proportion of non‐viable seeds from the pure agar treatment within each population (species × site) was the same across replicates and treatments because the seeds were randomly assigned to Petri dishes. Therefore, we only conducted the viability test on the seeds left in the Petri dishes from the pure agar treatment. We found that there was on average one non‐viable seed per Petri dish. To get a more accurate estimate of germination percentage, we, therefore, subtracted one seed from the total sum of seeds that could have germinated in each Petri dish, except for the Petri dishes where all seeds germinated. Using this method, we assumed that viable seeds that did not germinate died or were otherwise inhibited from germinating due to the drought treatment sometime during the experiment rather than due to an uneven distribution of viable seeds among Petri dishes from the start.

### 2.4 Trait measurements

We measured seed mass by drying seeds at 65°C for 48 h and weighing them in bulk to get the average seed mass per population (Pérez‐Harguindeguy et al., 2013). For each population, we weighed five samples of 50 seeds, from which we derived the average for that population. For *S. procumbens*, we did not have enough seeds to have five samples with 50 seeds from each population. Hence, for the population at 2130 mm/year, we had three samples of 50 seeds, and for the driest site (1226 mm/year), we had one sample with 50 seeds and one with 33 seeds.

When the seedlings had developed their first true leaf (*S. procumbens*)/pair of true leaves (*V. alpina*) as described above, we harvested them for measuring traits. We divided the plant into above‐ and belowground biomass and dried them in the oven for at least 48 h at 60°C and weighed each part individually to obtain dry mass (Pérez‐Harguindeguy et al., 2013). From this, we calculated three whole‐seedling traits: belowground dry mass, aboveground dry mass, and root–shoot ratio (dried belowground biomass/dried aboveground mass).

### 2.5 Data analysis

We constructed Bayesian models (Hobbs & Hooten, 2015) to test how the interactive effects of population and moisture treatment determine germination success. The dependent variables at the Petri dish level include germination percentage (number of seeds germinating/viable seeds), T_50_ (days to 50% germination), and days to max germination (number of days it takes to reach the maximum germination percentage). Germination percentage (*G*) was a binomially distributed variable tracked by the percent successful germinants ($\hat{G}$) per number of viable seeds (*V*) in a dish: $G∼\text{Binomial}\left(\hat{G},V\right)$. T_50_ and days until max germination are all over‐dispersed count data modeled as a negative binomial process, where days (*D*) have a mean ($\hat{D}$) and dispersion parameter, θ: $D∼\text{NegBinom}\left(\hat{D},\theta \right)$. The binomially distributed variables had a logit‐link function while the negative binomial variables had a log‐link function.

We used R (R Core Development Team, 2019) with Gibbs sampling implemented in JAGS (Plummer, 2003) with the “R2jags” package (Su & Yajima, 2015). Models for germination percentage and time to max germination ran for at least 100,000 iterations with a 35,000 iteration burn‐in and thinning rate of 5. Models for T_50_ and seedling traits ran for at least 50,000 iterations with a 15,000 iteration burn‐in and thinning rate of 5. Gelman–Rubin statistics were <1.05 for all variables in each model (Gelman & Rubin, 1992). We checked parameter trace plots to ensure good mixing and uniquely identified parameters. Further, we used two forms of posterior predictive checks to assess if the models were fitting properly. First, we assessed Bayesian “*p*‐values”, which take a discrepancy metric (i.e., the sum of squared residuals) and asked if data simulated from the model had a similar predictive error as the observed data (~0.5 indicates proper fit; Gelman et al., 1996). Second, we plotted the sum of squared residuals for the simulated against the observed data. All priors were vague (“flat”) with normal priors for slope and intercept terms describing treatment effects (*N*(0, 1E‐6)), gamma priors for random effect variance terms (Γ(0.001, 0.001)), and uniform distribution for the dispersion term of the negative binomial distribution (U(0,50)).

For max germination percentage models, Bayesian “*p*‐values” indicated that the model was overconfident (i.e., data simulated from the model had less error than the observed data). By investigating other diagnostics plots, we identified that this was due to a long tail of zero germination in the lowest water potentials, making it difficult to fit the model through water potentials where germination occurred. To improve model fit, we removed the long tail of zero germination at the low water potentials by excluding the drought treatments after the first water potential level with no germination. This did not improve the Bayesian “*p*‐values”, but other assessments of model fit (Gelman–Rubin test, trace plots, and residual checks) improved, so we proceeded with these models.

Similarly, for the seedling models, we only modeled the seedlings at −0.57 MPa or above. In *V. alpina* that meant removing eight data points, while for *S. procumbens* we removed 54 data points (where 52 of these data points are from the second driest population). This avoided forcing model fits at very dry treatments based on little information.

We addressed the three general research questions with specific predictions as follows:

1. Do populations of alpine species from climates and microhabitats reflecting different moisture regimes vary in their seed germination response to moisture availability?

Specifically, we tested the following predictions: (P1) We expect low moisture availability to negatively affect all germination metrics, and (P2) Seeds from drier climates will be less negatively affected by drought conditions than seeds from wetter climates, indicating local adaptation. Therefore, in separate models by species, we modeled the linear effect of water potential treatments (WP) interacting with the annual precipitation of the site (as a factor) on a given germination metric. Max germination percentage and days to max germination are simple linear regressions. Individual seed‐/germinant‐based dependent variables have Petri dish random effects in addition.

2 Are these patterns and responses also reflected in seedling growth and development traits?

For research question 2, we specifically tested the following predictions: (P3) Seedling biomass will decrease and root–shoot ratio will increase toward more intense drought, and (P4) seedlings from the driest populations will tolerate low water availability better (i.e., have larger seedlings and lower root–shoot ratio) than seedlings from wetter populations. We tested these predictions by modeling treatment effects on whole‐seedling growth and development traits using similar models as above, where each seedling is a replicate, with Petri dish as a random effect in separate models for each species. We analyzed how traits differ linearly by water potential treatment and investigated if the traits of populations differed among water potential treatments according to home site annual precipitation as in question 1, but with traits as the dependent variable. All traits, *T*, were log‐transformed to be normally distributed with a mean ($\hat{T}$) and variance (*σ*): $T∼N\left(\hat{T},\sigma \right)$. Variance terms (*σ*) for traits will have gamma prior distributions (Γ(0.001, 0.001)).

3 Are germination and growth responses to moisture availability dependent on seed mass?

For research question 3, we specifically tested the following prediction: (P5) Within each species, populations from drier habitats will produce larger seeds, leading to greater germination and seedling success overall, and especially so under drier conditions. First, we tested if seed size varies as predicted with site annual precipitation and species using a simple multiplicative generalized linear model with a gamma distribution, followed by a Tukey's post hoc test. Then, in models of questions 1 and 2, we replaced precipitation with seed mass across the populations as a numerical predictor variable, which allows us to test our prediction on how seed size affects germination and seedling traits.

Finally, linked to both questions 1 and 2, we expected stronger patterns in both germination and seedling traits across populations in the generalist species *V. alpina* compared to the snowbed specialist *S. procumbens* because less microhabitat selectivity locally exposes the populations more to the effects of regional climate (P6).

We measured water potentials that translates into water availability for the seeds/plants which could have implications for how plants respond to drought. In the Results, we describe the trends with water potentials, while in the Discussion we talk about how water availability impacts early life stages of alpine plants and how this could impact drought responses.

## 3 RESULTS

We report the effect of drought (lower water potentials), relative to the pure agar treatment (the highest water potential). Accordingly, the pure agar treatment is found on the right‐hand side of the figures, with increasing drought toward the left‐hand side.

**FIGURE 2:**
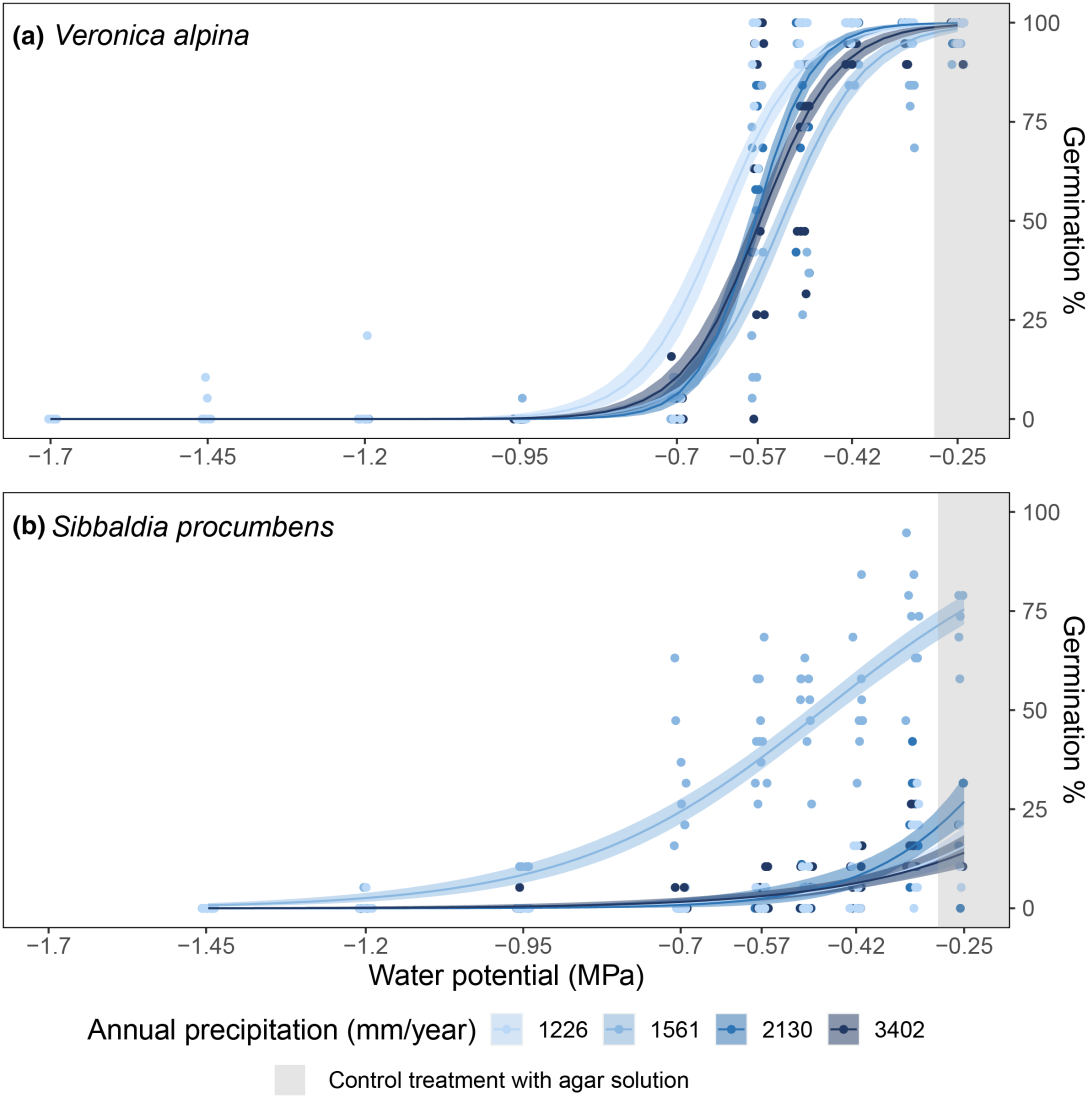
Germination percentage (number of seeds germinated/viable seeds in the Petri dish) for (a) *Veronica alpina* and (b) *Sibbaldia procumbens* across different water potentials (MPa). −0.25 MPa represents pure agar medium with no drought treatment (grey background color). Decreasing water potential yields increasing levels of drought. The colors are based on the average annual precipitation from 2009 to 2019 of the populations the seeds were sampled from, going from light blue in the driest to dark blue in the wettest population. Seeds were sampled at alpine sites in Western Norway. The lines represent predicted means from the model with 95% credible interval envelopes.

In total, 2998 of 7248 seeds and 780 of 6058 seeds germinated for *V. alpina* and *S. procumbens*, respectively (Table 1). In the three highest water potential treatments (down to −0.42 MPa), 95%–100% of *V. alpina* seeds germinated (Figure 2). The germination percentage then decreased rapidly from around 100% to around 0% between −0.50 and −0.70 MPa (Table 1, Figure 2). For *V. alpina*, 85%–95% of the seeds that germinated developed the first pair of true leaves at high water potentials, only 55% did so at −0.70 MPa, and none at the lowest water potentials (Table 1). Seeds from the driest population germinated at higher percentages in lower water potentials compared to the other three populations (Figure 2, Table 2). For *S. procumbens*, germination percentage also varied significantly between populations, but not in the expected patterns along the precipitation gradient (Figure 2, Table 2). In the pure agar treatment, the population from the second driest site reached up to 75% germination (Figure 2), whereas the other populations only reached about 20%–25% germination (Figure 2). In all *S. procumbens* populations, germination gradually decreased with decreasing water potentials until −0.95 MPa, where very few seeds germinated (Figure 2, Table 1). For *S. procumbens*, about 95%–100% of seeds that germinated developed fully past the cotyledon stage and made at least one true leaf down to −0.70 MPa, but that percentage decreased to around 50% at lower water potentials (Table 1).

**TABLE 1:** Number of seeds that germinated, and seedlings that developed past the cotyledon phase and developed true leaves, per water potential treatment for two species in germination under drought conditions experiment.

Water potential (MPa)	*Veronica alpina*			*Sibbaldia procumbens*		
	Seeds sowed	# germinated	# seedlings with true leaves	Seeds sowed	# germinated	# seedlings with true leaves
−0.25	720	679	626	720	185	185
−0.33	720	663	578	720	213	208
−0.42	719	673	597	540	111	111
−0.50	719	514	489	659	104	100
−0.57	740	443	413	699	103	88
−0.70	740	18	10	540	53	49
−0.95	725	1		540	9	5
−1.20	720	4		560	2	1
−1.45	720	3		540		
−1.70	725			540		
Total	7248	2998	2713	6058	780	747

*Note*: We sowed 7248 seeds of *Veronica alpina* and 6058 seeds of *Sibbaldia procumbens*.

**TABLE 2:** Bayesian model outputs assessing how water potential affects germination metrics and seedling traits from different populations across a precipitation gradient.

Variable	Germination percentage (%)							
	*Veronica alpina* (*n* = 285)				*Sibbaldia procumbens* (*n* = 271)			
	Median	Std.dev	Lower 95% CI	Upper 95% CI	Median	Std.dev	Lower 95% CI	Upper 95% CI
*Intercept – Prec1* (*1226 mm/year*)	*−0.134*	*0.120*	*−0.372*	*0.099*	**−4.062**	0.283	−4.666	−3.560
*Prec2* (*1561 mm/year*)	**−1.471**	0.189	−1.846	−1.108	**3.201**	0.293	2.678	3.821
*Prec3* (*2130 mm/year*)	**−1.253**	0.203	−1.663	−0.860	−0.250	0.595	−1.482	0.867
*Prec4* (*3402 mm/year*)	**−1.024**	0.181	−1.383	−0.672	0.230	0.383	−0.518	0.987
Water potential: Prec1	**5.564**	0.365	4.893	6.299	**2.137**	0.329	1.536	2.822
Water potential: Prec2	−0.168	0.483	−1.121	0.763	−0.351	0.345	−1.064	0.283
Water potential: Prec3	**2.232**	0.628	1.031	3.494	0.851	0.631	−0.356	2.126
Water potential: Prec4	0.225	0.512	−0.785	1.220	−0.320	0.450	−1.209	0.549
*R* ^2^	.75	.01	0.73	.77	.73	.02	.70	.76

Variable	Time to max germination (days)							
	*Veronica alpina* (*n* = 177)				*Sibbaldia procumbens* (*n* = 130)			
	Median	Std.dev	Lower 95% CI	Upper 95% CI	Median	Std.dev	Lower 95% CI	Upper 95% CI
*Intercept – Prec1* (*1226 mm/year*)	**2.684**	0.086	2.519	2.855	**4.428**	0.143	4.161	4.721
*Prec2* (*1561 mm/year*)	0.229	0.118	−0.003	0.459	0.064	0.184	−0.298	0.425
*Prec3* (*2130 mm/year*)	**0.724**	0.115	0.497	0.953	−0.017	0.260	−0.505	0.514
*Prec4* (*3402 mm/year*)	**0.672**	0.117	0.442	0.903	−0.226	0.195	−0.613	0.156
Water potential: Prec1	**−0.623**	0.085	−0.793	−0.457	0.202	0.154	−0.104	0.501
Water potential: Prec2	**0.257**	0.118	0.026	0.489	−0.048	0.194	−0.428	0.335
Water potential: Prec3	**0.304**	0.113	0.081	0.525	0.204	0.283	−0.379	0.733
Water potential: Prec4	0.064	0.123	−0.176	0.306	0.051	0.210	−0.360	0.461
Dispersion	4.306	0.551	3.356	5.508	1.763	0.219	1.376	2.227
*R* ^2^	.53	.04	.46	.60	.09	.03	.03	.16

Variable	Time to 50% germination (days)							
	*Veronica alpina* (*n* = 177)				*Sibbaldia procumbens* (*n* = 130)			
	Median	Std.dev	Lower 95% CI	Upper 95% CI	Median	Std.dev	Lower 95% CI	Upper 95% CI
*Intercept – Prec1* (*1226 mm/year*)	**1.727**	0.080	1.567	1.882	**4.038**	0.158	3.744	4.361
*Prec2* (*1561 mm/year*)	0.170	0.109	−0.042	0.383	**−2.141**	0.211	−2.565	−1.731
*Prec3* (*2130 mm/year*)	**0.373**	0.108	0.163	0.586	0.065	0.287	−0.468	0.660
*Prec4* (*3402 mm/year*)	**0.500**	0.105	0.295	0.709	**−0.443**	0.217	−0.874	−0.020
Water potential: Prec1	**−0.549**	0.080	−0.707	−0.394	−0.020	0.161	−0.340	0.296
Water potential: Prec2	0.048	0.108	−0.161	0.260	−0.090	0.208	−0.497	0.319
Water potential: Prec3	−0.195	0.106	−0.402	0.014	0.225	0.299	−0.389	0.784
Water potential: Prec4	−0.091	0.106	−0.297	0.118	−0.086	0.226	−0.533	0.358
Dispersion	13.950	3.822	8.988	23.939	1.442	0.178	1.125	1.823
*R* ^2^	.75	.01	.72	.77	.49	.03	.41	.55

Variable	Belowground biomass (g)							
	*Veronica alpina* (*n* = 2653)				*Sibbaldia procumbens* (*n* = 727)			
	Median	Std.dev	Lower 95% CI	Upper 95% CI	Median	Std.dev	Lower 95% CI	Upper 95% CI
*Intercept – Prec1* (*1226 mm/year*)	**−8.996**	0.083	−9.159	−8.834	**−9.793**	0.155	−10.094	−9.490
*Prec2* (*1561 mm/year*)	−0.219	0.120	−0.451	0.019	0.042	0.163	−0.275	0.366
*Prec3* (*2130 mm/year*)	−0.225	0.117	−0.457	0.004	−0.113	0.276	−0.661	0.427
*Prec4* (*3402 mm/year*)	**−0.284**	0.118	−0.516	−0.051	**0.435**	0.217	0.009	0.859
Water potential: Prec1	**−0.512**	0.081	−0.671	−0.354	**0.559**	0.206	0.154	0.962
Water potential: Prec2	−0.122	0.120	−0.354	0.117	**−0.651**	0.212	−1.072	−0.236
Water potential: Prec3	0.072	0.114	−0.153	0.297	**−0.805**	0.333	−1.454	−0.147
Water potential: Prec4	0.109	0.116	−0.118	0.335	**−0.566**	0.263	−1.077	−0.050
Variance of trait	1.058	0.015	1.028	1.087	1.108	0.030	1.051	1.170
Variance random effect	0.481	0.036	0.415	0.556	0.099	0.064	0.026	0.258
*R* ^2^	.17	.02	.13	.22	.05	.01	.02	.08

Variable	Aboveground biomass (g)							
	*Veronica alpina* (*n* = 2672)				*Sibbaldia procumbens* (*n* = 736)			
	Median	Std.dev	Lower 95% CI	Upper 95% CI	Median	Std.dev	Lower 95% CI	Upper 95% CI
*Intercept – Prec1* (*1226 mm/year*)	**−9.072**	0.042	−9.152	−8.988	**−8.215**	0.092	−8.395	−8.035
*Prec2* (*1561 mm/year*)	−0.015	0.061	−0.136	0.104	0.099	0.097	−0.092	0.291
*Prec3* (*2130 mm/year*)	−0.018	0.059	−0.136	0.098	0.135	0.160	−0.180	0.452
*Prec4* (*3402 mm/year*)	−0.014	0.060	−0.132	0.104	**0.296**	0.128	0.045	0.547
Water potential: Prec1	−0.071	0.041	−0.151	0.010	0.021	0.120	−0.215	0.256
Water potential: Prec2	**−0.252**	0.061	−0.372	−0.132	−0.033	0.124	−0.278	0.213
Water potential: Prec3	**−0.133**	0.059	−0.248	−0.019	−0.274	0.194	−0.651	0.108
Water potential: Prec4	**−0.134**	0.060	−0.251	−0.018	0.149	0.154	−0.147	0.453
Variance of trait	0.752	0.011	0.731	0.773	**0.637**	0.018	0.603	0.673
Variance random effect	0.203	0.021	0.164	0.247	**0.122**	0.042	0.038	0.202
*R* ^2^	.07	.01	.05	.10	.02	.01	.05	.03

Variable	Root–shoot ratio (g/g)							
	*Veronica alpina* (*n* = 2653)				*Sibbaldia procumbens* (*n* = 727)			
	Median	Std.dev	Lower 95% CI	Upper 95% CI	Median	Std.dev	Lower 95% CI	Upper 95% CI
*Intercept – Prec1* (*1226 mm/year*)	0.071	0.073	−0.071	0.215	**−1.575**	0.146	−1.865	−1.289
*Prec2* (*1561 mm/year*)	**−0.218**	0.106	−0.428	−0.010	−0.061	0.155	−0.363	0.247
*Prec3* (*2130 mm/year*)	−0.200	0.104	−0.405	0.005	−0.318	0.261	−0.832	0.183
*Prec4* (*3402 mm/year*)	**−0.281**	0.104	−0.487	−0.080	0.141	0.206	−0.264	0.549
Water potential: Prec1	**−0.438**	0.071	−0.577	−0.299	**0.541**	0.194	0.156	0.927
Water potential: Prec2	0.111	0.106	−0.096	0.317	**−0.620**	0.200	−1.011	−0.224
Water potential: Prec3	0.197	0.102	−0.003	0.396	−0.492	0.315	−1.103	0.124
Water potential: Prec4	**0.248**	0.103	0.047	0.450	**−0.725**	0.249	−1.207	−0.233
Variance of trait	1.032	0.015	1.003	1.061	1.043	0.029	0.987	1.102
Variance random effect	0.407	0.033	0.347	0.476	0.117	0.069	0.027	0.276
*R* ^2^	.09	.02	.06	.13	.04	.01	.01	.06

*Note*: For each model, the water potential of each observation was standardized by subtracting the mean water potential and dividing it by the standard deviation. Each number is the model estimate based on one unit of the standardized water potential and indicates the effect of decreasing drought (going from low to high water potential). Bolded numbers in the median column are those where the 95% credible interval (CI) does not overlap 0, indicating the significance level with a threshold of 0.05 in a frequentist framework. The populations come from different precipitation levels, going from 1226 mm/year in the driest site (Prec1) to 1561 mm/year (Prec2), 2130 mm/year (Prec3), and finally, 3402 mm/year in the wettest site (Prec4). Seeds were sampled at alpine sites in Western Norway. Sample size of the germination traits is number of Petri dishes while the germination traits use individual seedlings, hence, the difference in sample size between models.

**FIGURE 3:**
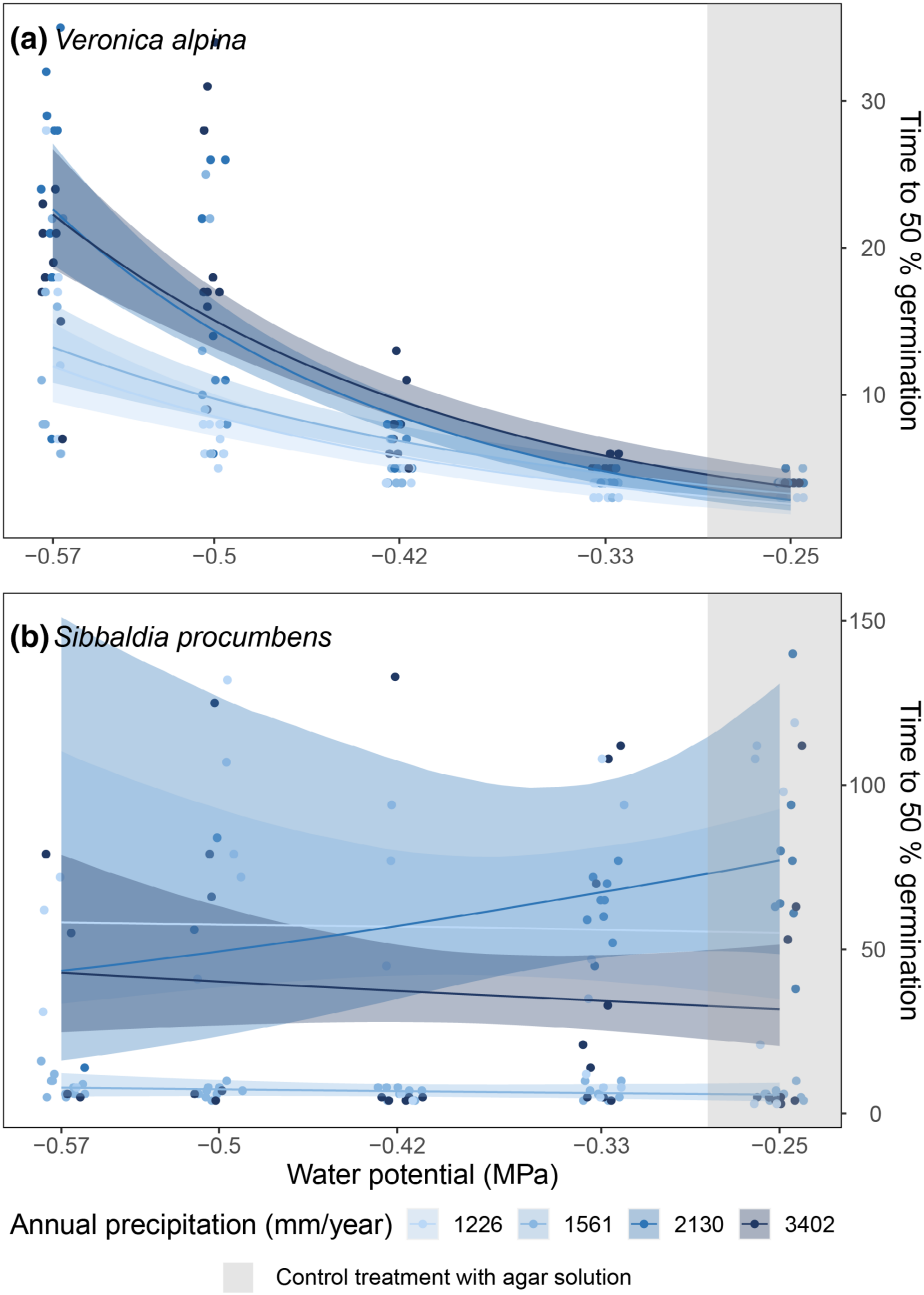
Time to 50% germination (in days) for (a) *Veronica alpina* and (b) *Sibbaldia procumbens* across different water potentials (MPa). Note the different scales on the y‐axis. −0.25 MPa represents pure agar medium with no drought treatment (grey background color). The decreasing water potential yields increasing levels of drought. The colors are based on the average annual precipitation from 2009 to 2019 of the populations the seeds were sampled from, going from light blue in the driest to dark blue in the wettest population. Seeds were sampled at alpine sites in Western Norway. The lines represent predicted means with 95% credible interval envelopes.

In the pure agar treatment, it took on average of 4 days for *V. alpina* to reach 50% germination (Figure 3). Germination rates decreased significantly with decreasing water potentials, especially in populations from wetter sites (Figure 3; Table 2). The two driest populations reached 50% germination at −0.57 MPa after approximately 15 days, and for the two wettest populations, it took 20 days (Figure 3). Time to max germination also increased significantly with decreasing water potential, and at a faster rate in the driest and the wettest populations (Table 2). For *S. procumbens*, it took on average 66 days to reach 50% germination in the pure agar treatment in the population from the driest site, 6 days in the second driest population, 77 days in the second wettest population, and 28 days for the wettest population (Figure 3). There was no systematic or significant change in time to 50% germination or the time to max germination with water potential for *S. procumbens*, and there were also no interactions between water potential and populations (Table 2). For *V. alpina*, there was also a decrease in synchrony between the different replicates as water potential decreased (Appendix S1: Figure A.1), while for *S. procumbens*, there was no obvious trend (Appendix S1: Figure A.2).

**FIGURE 4:**
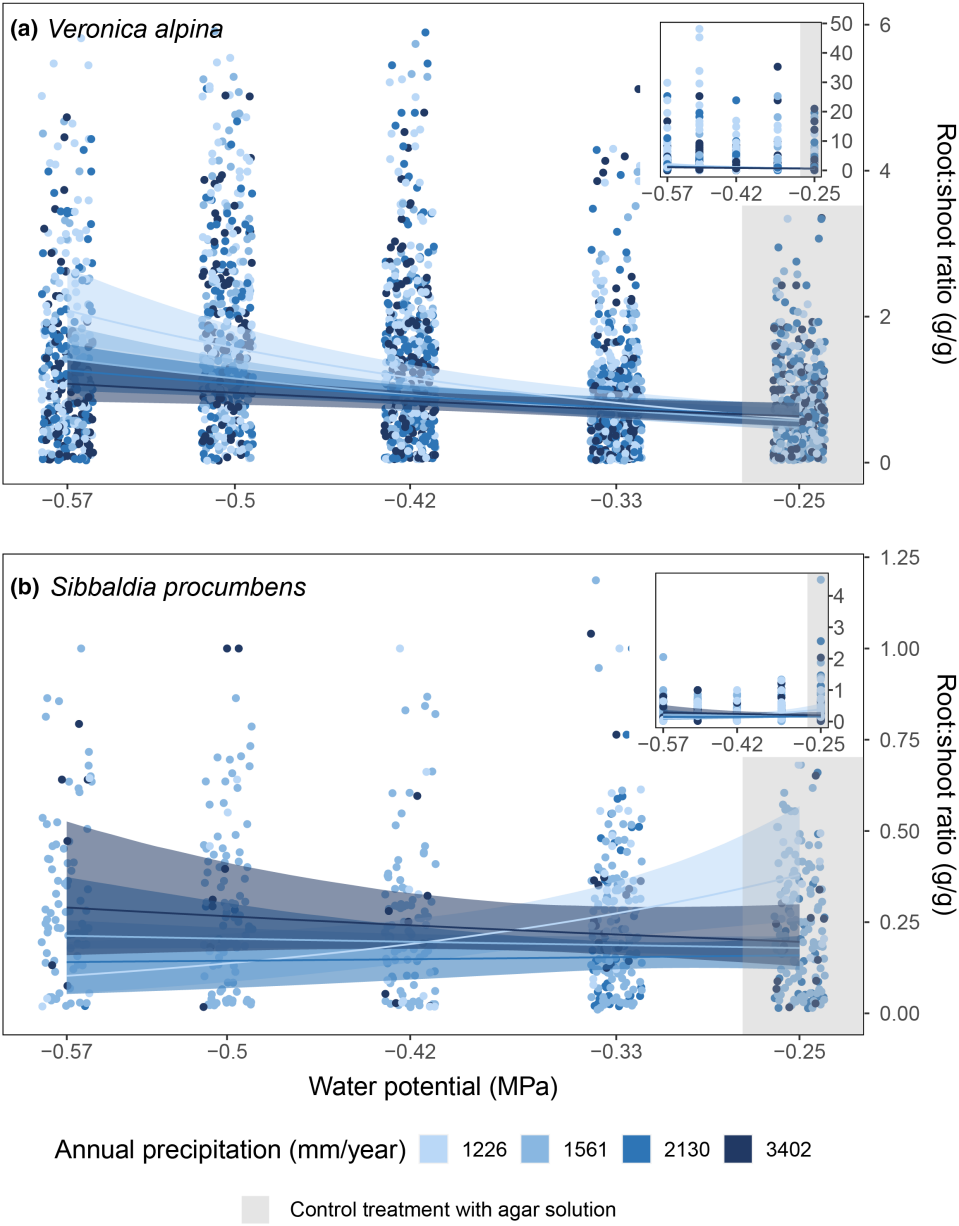
Root–shoot ratio for (a) *Veronica alpina* and (b) *Sibbaldia procumbens* across different water potentials (MPa). Note the different scales on the y‐axis. −0.25 MPa represents pure agar medium with no drought treatment/grey backgorund color). The inserts show all the data (the models are built on all the data), while the large panels zoom in on the patterns in the data excluding some outliers (showing 96% of the data for *V. alpina* and 98% for *S. procumbens*). The colors are based on the average annual precipitation from 2009 to 2019 of the populations the seeds were sampled from, going from light blue in the driest to dark blue in the wettest population. Seeds were sampled at alpine sites in Western Norway. The lines represent predicted means with 95% credible interval envelopes.

The average seedling mass in the pure agar treatment of *V. alpina* and *S. procumbens* was 0.167 and 0.410 mg, and the average root–shoot ratio was 0.789 and 0.328, respectively. Seedlings of *V. alpina* had higher root–shoot ratios in the lowest water potentials with an average of 2.03, and significantly more so in seedlings from the population from the driest site, 3.36 compared to 1.35 on average across the other three populations (Figure 4, Table 2). The difference in root–shoot ratio in *V. alpina* was driven by higher belowground biomass production as the aboveground biomass stayed relatively constant across populations and water potentials (Table 2). The seedling biomass of *S. procumbens* did not vary systematically either between populations or across water potentials (Figure 4, Table 2).

**FIGURE 5:**
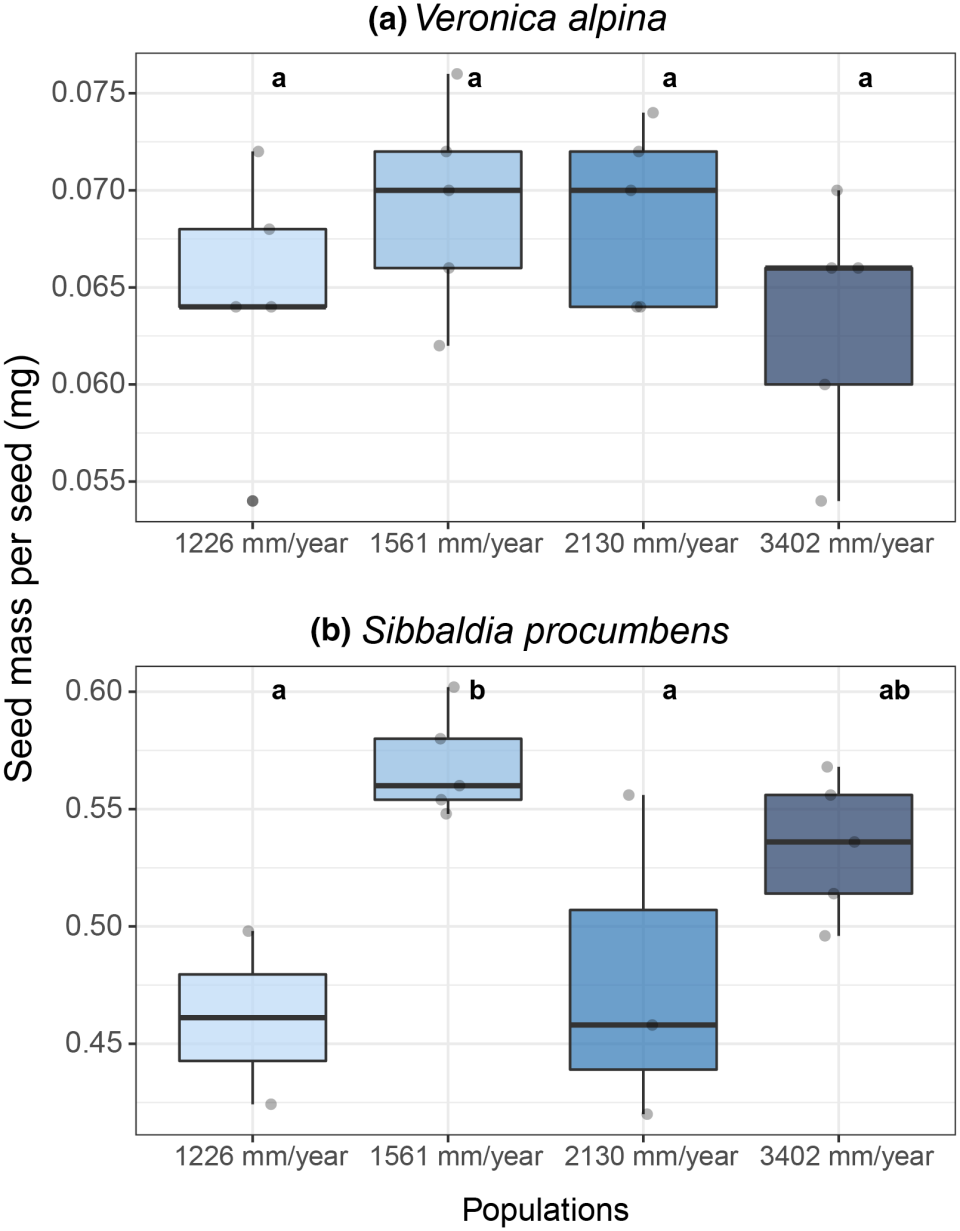
Seed mass per seed for (a) *Veronica alpina* and (b) *Sibbaldia procumbens* from populations across a precipitation gradient. The colors are based on the average annual precipitation from 2009 to 2019 of the populations the seeds were sampled from, going from light blue in the driest to dark blue in the wettest population. Seeds were sampled at alpine sites in Western Norway. Note the different scales on the y‐axis. Letters are based on significant differences between populations in a generalized linear model.

The average seed mass for *V. alpina* and *S. procumbens* was 0.0664 and 0.525 mg, respectively. For *V. alpina*, there was no significant variation in seed mass between populations (Figure 5). In *S. procumbens*, the second driest population had significantly heavier seeds than the driest and the second wettest populations (Figure 5) (*p*‐value < .03).

**FIGURE 6:**
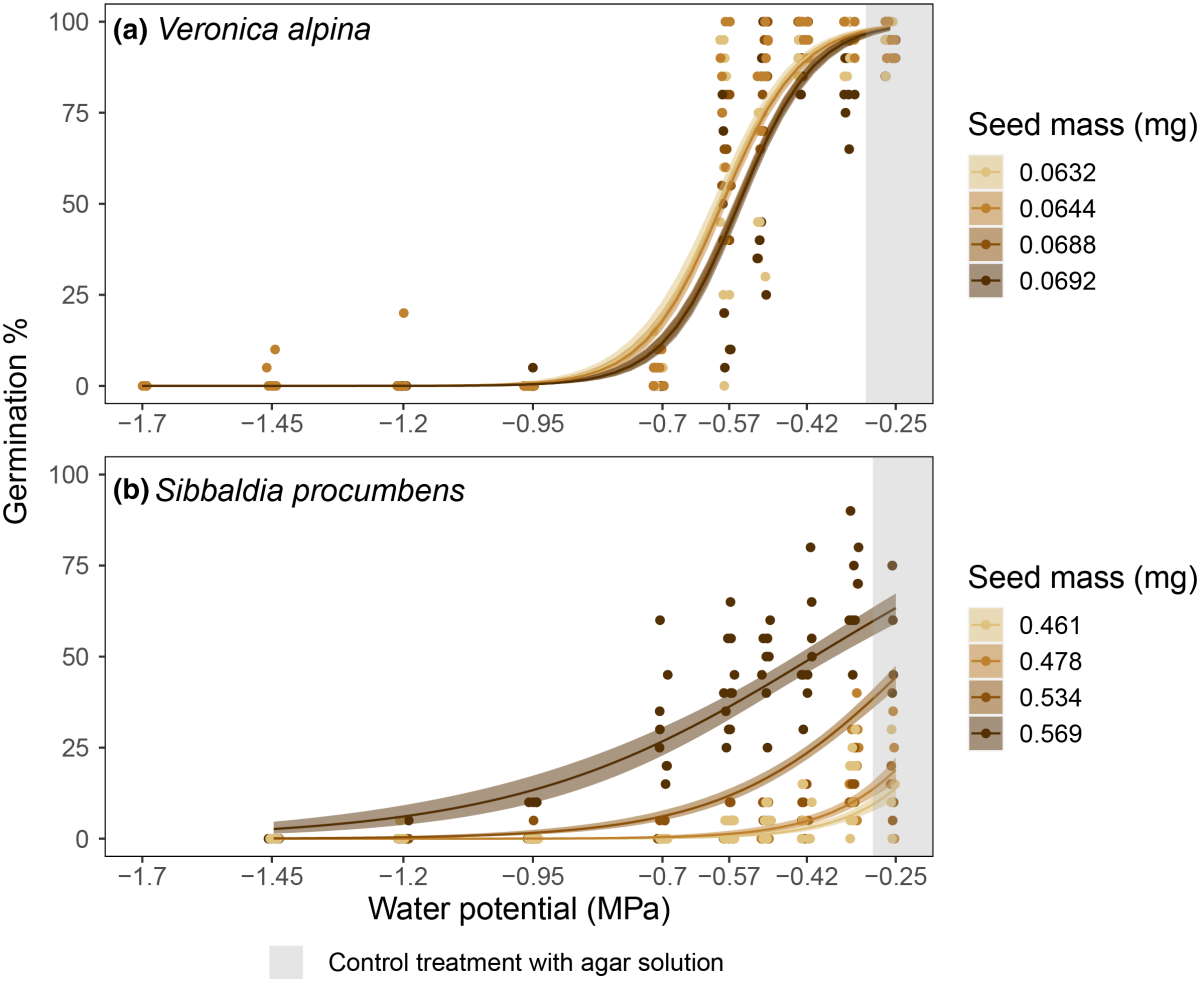
Germination percentage (number of seeds germinated/viable seeds in the Petri dish) for (a) *Veronica alpina* and (b) *Sibbaldia procumbens* across different water potentials (MPa). −0.25 MPa represents pure agar medium with no drought treatment (grey background color). The decreasing water potential yields increasing levels of drought. The colors are based on the seed mass of the different populations within each species, where dark brown represents the population with the heaviest seeds and beige the population with the lightest seeds (note the difference in seed size between species). Seeds were sampled at four alpine sites in Western Norway, across a large precipitation gradient. Matching the seed mass to precipitation for *V. alpina*, the population with 0.0632 mg seed mass comes from the population with an annual precipitation average between 2009 and 2019 of 3402 mm/year, 0.0644 mg from 1226 mm/year, 0.0688 mg from 2130 mm/year and 0.0692 mg from the population with 1561 mm/year. For *S. procumbens*, the population with seeds of 0.461 mg comes from the population at 1226 mm/year, 0.478 mg from 2130 mm/year, 0.534 mg from 3402 mm/year, and 0.569 mg from 1561 mm/year. The lines represent predicted means with 95% credible interval envelopes.

For *V. alpina*, seed mass negatively affected germination percentage so that the lighter‐seeded populations germinated at higher percentages in general, but also with decreasing water potential (Figure 6, Appendix S2: Table A2.1). While seed mass did not affect any of the other germination responses or seedling traits directly, there was an interaction between seed mass and water potential in time to max germination and aboveground biomass (Appendix S2: Table A2.1, Figure A2.1, A2.2). For time to max germination, the lighter‐seeded populations took longer to germinate with decreasing water potentials than the heavier‐seeded populations (Appendix S2: Table A2.1, Figure A2.1). Additionally, the drought‐induced increase in aboveground biomass was more prominent in the heavier‐seeded populations (Appendix S2: Table A2.1, Figure A2.2).

Seed mass significantly affected germination percentage in *S. procumbens*, where the population with the heaviest seeds germinated at a higher percentage overall (Figure 6, Appendix S2: Table A2.1). The heavier‐seeded populations also had the least decline in germination percentage with decreasing water potential (smaller slopes in Figure 6, Appendix S2: Table A2.1). Time to 50% germination is also affected by seed mass as lighter‐seeded populations take longer to germinate than the heavier‐seeded populations (Appendix S2: Table A2.1, Figure A2.3).

## 4 DISCUSSION

Water availability affected the seed regeneration of both alpine forb species. Germination percentage dropped sharply below a certain water potential for *V. alpina*, while declining gradually with decreasing water potential for *S. procumbens*. Low water availability also led to delayed germination and a higher investment in roots in the surviving seedlings for *V. alpina*. We found that these germination and seedling responses to low water availability in *V. alpina* were stronger in individuals from the driest populations, which could indicate that these populations are locally adapted to a drier climate, where intermittent droughts are more likely to occur. In addition to precipitation of the source site, seed mass was also important in describing germination patterns for *V. alpina*, where populations with lighter seeds reached higher germination percentages but had slower germination rates compared to heavier‐seeded populations. For *S. procumbens*, we did not find evidence of any responses to low water availability other than reduced germination percentage. The precipitation level of the source site also did not affect any germination responses or seedling traits in *S. procumbens*; instead, seed mass was more important as seeds from populations with higher seed mass germinated at higher percentages and faster rates. Overall, these contrasting results between species in early life‐history characteristics may be related to their different niches as an alpine generalist (*V. alpina*) and a snowbed specialist (*S. procumbens*). We discuss these findings in order where we start off by explaining the responses to drought in germination metrics (P1) and seedling traits (P3), followed by a discussion of potential explanations for adaptations to drought starting with seed mass (P5), local adaptation from the population's precipitation level (P2 and P4), and finally, the difference between species niches (P6).

### 4.1 Drought impacts on germination – Physiology and dormancy

Fewer seeds germinated in both species as water availability decreased, and seeds from *V. alpina* also took longer to germinate under low water availability in line with findings of a recent global meta‐analysis of alpine seeds and seedlings (Vázquez‐Ramírez & Venn, 2021). These findings are in line with our prediction that germination is negatively affected by low water availability across multiple metrics (P1). This indicates that low moisture availability leads to partial or complete inhibition of physiological processes which may slow down the germination processes or hinder seeds from germinating (Baskin & Baskin, 2014; Sumner & Venn, 2021). Further, we found a sharp decrease in germination percentage for *V. alpina* when water potential reaches between −0.57 and − 0.70 MPa. Such abrupt responses in germination to relatively minor shifts in the environment could be indicative of primary or secondary dormancy; in our case, water availability threshold or other dormancy release cues related to soil moisture preventing seeds from germinating under conditions where they are physiologically able to (Baskin & Baskin, 2014; Buijs, 2020; Jurado & Flores, 2005; Vleeshouwers et al., 1995). Many species from drought‐prone climates are known to have dormancy strategies that prevent germination from occurring during drought (Jurado & Flores, 2005), but to our knowledge, such responses have not been reported in systems as wet as ours. One potential mechanism could be physical dormancy due to low seed coat permeability, hindering seeds from taking up water and thus germinating at low water potentials (Baskin & Baskin, 2014; Saatkamp et al., 2019). Additionally, low germination at low water availability could be due to seed mortality (Baskin & Baskin, 2014). For *S. procumbens*, we find no threshold for germination percentage, or a systematic change in T_50_ or time to max germination in response to water availability. Hence, for both species, we find that drought reduced physiological processes and therefore germination success, while for *V. alpina*, we also identified a water potential threshold that could indicate a dormancy strategy to avoid germinating under low water availability.

### 4.2 Drought impacts on seedling strategies

For both species, seedling survival declined with decreasing water availability in line with results from other studies (Everingham et al., 2021; Harrison & LaForgia, 2019; Hovenden et al., 2008). Surviving seedlings of *V. alpina* showed a higher investment in belowground biomass at low water availability in line with our prediction (P3) and other studies (Harrison & LaForgia, 2019; Larson et al., 2020). This suggests an optimized resource allocation strategy where biomass investment is made in organs that increase the uptake of the limiting resource (Bloom et al., 1985); in this case, roots for water uptake rather than light and carbon gain through acquisitive leaf traits (Harrison & LaForgia, 2019). We found no systematic change in aboveground biomass with drought for either of our species. This could indicate that the increased belowground biomass in *V. alpina* seedlings may acquire sufficient moisture to maintain aboveground biomass production. On the other hand, *S. procumbens* seedlings did not show any differentiation in seedling biomass allocation across the water availability gradient, indicating a lack of drought strategies in the seedling stage, contradicting our prediction (P4).

### 4.3 Seed mass as a predictor of germination success

Our prediction that seeds from drier populations have heavier seeds (P5), as found in previous literature (Buckley, 1982; Leishman & Westoby, 1994; Moles & Westoby, 2004; Wellstein et al., 2013; Wright & Westoby, 1999) was not confirmed. Specifically, for *V. alpina*, we found no significant difference between the seed mass of the populations, and for *S. procumbens*, the significant difference in seed mass between populations was not related to precipitation. The seed mass of individuals and populations may vary as a result of many different environmental cues other than precipitation, such as temperature, solar radiation, seasonality of precipitation, or growing season length, among others (Galen & Stanton, 1993; Murray et al., 2004; Veselá et al., 2020). Some studies have found that seed mass increases with precipitation (Moles et al., 2005; Sandel et al., 2010), opposite to our prediction indicate more complex patterns with precipitation. In fact, precipitation has been proposed to be a poor predictor of seed mass compared to other more important factors such as temperature and solar radiation (Murray et al., 2004). These results suggest that precipitation level has not affected seed mass or the seed mass's role in promoting germination under drought for these species.

Even if seed mass does not correlate with precipitation in this study, seed mass can still be a good predictor for germination and seedling success with higher success in heavier‐seeded individuals under drought conditions (Jurado & Flores, 2005; Moles & Westoby, 2004; Westoby et al., 1996). However, we found mixed evidence for this prediction (P5). First, surprisingly, the lighter‐seeded species *V. alpina* germinated at higher percentages with low water availability compared to the larger‐seeded species *S. procumbens*. Second, within *V. alpina*, lighter‐seeded populations had higher germination percentages and fewer days to max germination than the heavier‐seeded populations. In contrast, we did find support for our prediction (P5) with *S. procumbens* where the heavier‐seeded population has the highest germination percentage and has the shortest time to 50% germination. Hence, we find support for the general pattern that heavier‐seeded species and populations generally tolerate low water availability better than lighter‐seeded seedlings in one of the two species for germination percentage and rate.

Dormancy is another important aspect of germination under low water potentials, which is more prevalent in lighter‐seeded species (Jurado & Flores, 2005; Thompson & Grime, 1979). As discussed above, our results could imply that the lighter‐seeded *V. alpina* may possess a dormancy strategy requiring high water availability in order to be broken, while no such germination threshold with water potential is present in the heavier‐seeded *S. procumbens*. This dormancy strategy might be enough to counteract the negative effects of drought during the early‐life stages, meaning other adaptations might not be needed. Additionally, larger‐seeded species generally have higher seedling survival and establishment success with lower water availability (Evans & Etherington, 1990; Leishman & Westoby, 1994). Our results align with this, with higher seedling survival in the larger‐seeded *S. procumbens* compared to *V. alpina* and increased aboveground biomass in the larger‐seeded population of *V. alpina* from the drier sites. However, as we were not able to measure seed mass on an individual level, there might be some links between germination responses and seedling traits to drought and seed mass that we are not able to identify in this study.

### 4.4 Drought strategies along a precipitation gradient in a high precipitation system

In this study, we are the first to identify that alpine species from very wet habitats (up to 3400 mm/year) can germinate under quite low water availability. The water potential threshold of *V. alpina* and the limit at which *S. procumbens* reaches around 0% germination is around −0.70 MPa. This water potential is described by Evans and Etherington (1990) as continuous water stress, representative of an intermediate dry environment before rainfall, or according to Young and Nobel (1986), corresponding to approximately 600 mm of rainfall. These descriptions correspond well to the average precipitation levels from 1960 to 1990 of the driest site in our precipitation gradient (Klanderud et al., 2015; Vandvik et al., 2020), indicating that the germination responses to low water availability might be adaptations to the historically drier precipitation levels. The observed minimum water potential for both species to germinate matches quite well with species from other moist climate regions such as England (Evans & Etherington, 1990), and in drier regions but alpine areas (Orsenigo et al., 2015). There are few studies on germination responses to drought that investigate populations from systems as wet as ours, with up to 3400 mm/year at the upper limit of our precipitation gradient. Yet, in one study of populations of *Calluna vulgaris* from coastal heathlands where the parent populations received annual precipitation between 1200 and 2000 mm/year, germination was more sensitive to drought than the two alpine species in this study (Birkeli et al., 2021). As plants respond predominantly to soil water availability rather than precipitation events themselves, drought responses may be driven by different factors. For example, an important aspect that varies between our study and Birkeli et al. (2021) is the higher salinity found in the coastal habitats of the parent generations of Birkeli et al.'s (2021) study, which has been found to be linked to germination success at low water availability (cf. Elnaggar et al., 2019). Overall, *V. alpina* and *S. procumbens* have the potential to germinate in conditions with quite low water availability, comparable to studies from drier habitats, despite coming from habitats with very high precipitation and soil moisture.

In one of our species, *V. alpina*, we found some evidence that seeds and seedlings from drier populations were more tolerant to lower water availability, consistent with our predictions (P2 and P4). The drier populations of *V. alpina* performed better than the other populations in terms of increased germination percentage, faster germination, and more allocation to roots when grown with lower water availability. These results may indicate that the populations from drier habitats are locally adapted to low water availability. Local adaptation to climatic conditions has also been found in other species from the same study system (Veselá et al., 2021). Whether the intraspecific variation in water availability responses we found is due to genetic differentiation or plasticity and, ultimately, relates to fitness requires further examination. For example, Veselá et al. (2021) found that three generations of greenhouse environment removed some of the adaptation to extreme conditions, suggesting that the germination responses are mostly plastic and not genetic. However, even if these local responses to water availability are only due to plasticity, Radersma et al. (2020) suggest that plasticity is an important driver of local adaptation and could lead to better‐adapted populations through genetic changes in the future. Similar evidence of better adaptation to drought in germination responses in the driest populations has been found in other systems (Cochrane et al., 2014; Torres‐Martínez et al., 2017), but these habitats are even drier than the driest site in our study. To our knowledge, no other studies have investigated the local adaptation to low water availability in germination and seedling traits in populations from sites with precipitation large and as wet as ours. Hence, in this study, we fill a knowledge gap by identifying local adaptation to low water availability even in populations from climates that are not traditionally characterized by drought due to very high annual precipitation.

Although the driest population does have a clear and significant difference from the other populations, it was not always the case that the wettest populations were the one that did the worst under the driest conditions. If there were in fact a linear trend, adding more sites along this precipitation could have helped to identify this trend. It could also indicate a potentially non‐linear trend along the precipitation gradient where other factors than pure precipitation amount might influence the tolerance to drought. Mean annual precipitation has been found to be less important for changes in germination and seedling traits, while climatic effects such as the duration of heatwaves and dry spells, or the range of precipitation were found to be more important (Everingham et al., 2021; Orsenigo et al., 2014). In our study system, the variation in precipitation is highest in the wettest end of the gradient (Gya, 2022), which could mean that the high variation in precipitation in the wettest sites could also increase the number of drought events for the wettest populations. High variation in precipitation has in fact been found to be linked to higher seed dormancy (Torres‐Martínez et al., 2017). In alpine systems such as ours, precipitation occurring as snow also play an important role in germination, with variation in snow depth, the timing of snow melt‐out, and water run‐off found to be important (Briceño et al., 2015; Hülber et al., 2011). Ultimately, our results indicate that for *V. alpina*, the driest population seems to be best adapted to low water potentials, but the lack of a clear linear trend with precipitation indicates that there might be other climatic or biotic factors that impact the ability to germinate at low water potentials.

### 4.5 Microhabitat selectivity as a predictor for drought tolerance

Within a site, different microhabitats may also influence germination success and seedling development. Alpine generalists have to be adapted to many different stressors of all the different microhabitats they grow in, such as producing seeds with dormancy to avoid germinating as water availability is low (Jurado & Flores, 2005). The high water availability and less frequent drought in snowbed habitats could mean that snowbed species experience little selection pressure for adaptations to low water availability. Our results support this by finding evidence of a water potential threshold, and local adaptation to low water availability in the seeds from the driest population in the alpine generalist (*V. alpina*) but not the snowbed specialist (*S. procumbens*), supporting our prediction (P6). Further, the lower germination percentage for *S. procumbens* that is more spread out in time (see Appendix S1: Figure A.1) could be an indication of bet hedging – spreading the germination out in time, a strategy well known for germination in habitats with high variability in climate (Evans & Dennehy, 2005). Bet hedging has been found to be a strategy that yields advantages during drought events (Evans & Dennehy, 2005; Lampei et al., 2017). A bet‐hedging strategy could be an advantage for snowbed species, such as *S. procumbens*, due to the unpredictable conditions in the snowbeds from year to year. Previous literature also suggests that under drought conditions, compared to generalists, snowbed species produce fewer seeds (Kudo & Hirao, 2006; Tonin et al., 2019) and have higher mortality in adult individuals (Oberbauer & Billings, 1981). Although our results are only based on two species, these results, combined with the existing literature, hint at an interesting difference between alpine generalists and snowbed specialists. Further empirical tests remain to confirm if there is a general pattern where snowbed species are more sensitive to drought than alpine generalist species.

### 4.6 Could local adaptation make populations better fit to face climate change?

Our results indicate that as alpine habitats are increasingly more exposed to drought events, some alpine species, or populations therein, may have the capacity to cope with these changes, while others have fewer of these adaptations. Species that have more plastic responses to drought require less genetic modification to lead to local adaptation (Radersma et al., 2020). In this study, increased plastic responses to low water availability suggest that *V. alpina* might have a quicker way to develop optimal germination strategies under drought conditions through local adaptation. In a much drier region, in Australia, Everingham et al. (2021) found that species had adapted to increasing drought events over time in both germination and seedling traits, indicating that species might be able to adapt at a fast rate to increasing drought. However, the species from Everingham et al. (2021) were all from systems that had already experienced drought and might have already had physiological or morphological traits for drought tolerance that evolution could act on. In this study, we find that the generalist species (*V. alpina*) has some of these physiological and morphological traits, while the snowbed specialist (*S. procumbens*) does not. Baskin and Baskin (2014) reviewed global data and found that regions that had consistently high precipitation over a longer period of time had lower dormancy than species from more drought‐exposed areas. Regions with consistently high precipitation over a longer period such as Norway might therefore have more species like *S. procumbens* where there are no, or few, physiological or morphological drought tolerance mechanisms for evolution to act upon. This in turn might mean that species and/or populations from habitats where water availability is currently high, and specifically, species that do not grow in microhabitats that experience drought events, might be extra sensitive to increased drought in the future.

### 4.7 Methodological remarks

Although germination rates from one *S. procumbens* population were much higher than the others, we do not believe that seed quality is a contributing factor. Low germination commonly arises if sampled seeds are not fully matured, or if dormancy restrictions are not broken. In our study, all seeds were sampled within the same time period, and we strived to only collect seeds that were mature at the time. As for dormancy breakage through cold stratification, all populations were treated for 9 weeks at 4°C, in a moist and dark environment. We believe that this is long enough to break dormancy because we observed the same higher germination percentage in one population when we sowed seeds from the same batch of seeds in the field for another study (R. Gya, V. Vandvik, unpublished data). These seeds were sowed out in the fall and got a full proper winter in the field, hence, that natural cold stratification would have broken the dormancy for all populations. Additionally, the range of germination percentage is within the ranges of other germination trials with *S. procumbens* (Graae et al., 2011; Varga & Kytöviita, 2016). All these arguments strengthen our confidence that the low germination percentage in three of the populations and the difference between populations in *S. procumbens* are not due to methodological errors. Instead, our results indicate that seed mass might by a driving component of this difference between populations rather than local adaptation along the precipitation gradient.

## 5 CONCLUSIONS

This study provides some of the first evidence that species from very wet alpine habitats can germinate under surprisingly dry conditions. One of the species, an alpine generalist, may have physiological (dormancy and fast germination) and morphological (higher root:shoot ratio) adaptations to drought, which are more pronounced in populations from drier habitats, confirming our predictions (P1, P2, P3, and P4). In contrast, the other species, a snowbed specialist, did not show evidence of potential drought adaptation, contradicting our predictions (P2, P3, and P4), but seed mass seemed to be important for germination success (confirming P5). These results indicate that some alpine species from high precipitation habitats could already have strategies in place to survive a climate with more drought, whereas others lack physiological and morphological traits to handle drought for evolution to act upon, making them more vulnerable to the future increase in drought events. Further investigation is needed to see if the results from this study, that generalist species are better adapted to drought due to more varying microclimates compared to the snowbed specialist (confirming P6), could be generalized beyond these two species. Both the inter‐ and intraspecific variation in seedling recruitment reported here may thus hint at which species and populations of alpine plants would suffer or thrive in a changing climate.

## AUTHOR CONTRIBUTIONS

**Ragnhild Gya:** Conceptualization (lead); data curation (lead); formal analysis (lead); investigation (lead); methodology (lead); project administration (lead); visualization (lead); writing – original draft (lead); writing – review and editing (lead). **Sonya Rita Geange:** Conceptualization (supporting); investigation (supporting); methodology (supporting); supervision (supporting); writing – original draft (supporting); writing – review and editing (equal). **Joshua Scott Lynn:** Conceptualization (supporting); formal analysis (supporting); investigation (supporting); methodology (supporting); supervision (supporting); writing – original draft (supporting); writing – review and editing (equal). **Joachim Paul Töpper:** Conceptualization (supporting); formal analysis (supporting); funding acquisition (lead); investigation (supporting); methodology (supporting); supervision (lead); writing – original draft (supporting); writing – review and editing (equal). **Øystein Wallevik:** Investigation (equal); writing – review and editing (equal). **Camilla Zernichow:** Investigation (equal); writing – review and editing (equal). **Vigdis Vandvik:** Conceptualization (supporting); funding acquisition (lead); methodology (supporting); supervision (lead); writing – original draft (supporting); writing – review and editing (equal).

## FUNDING INFORMATION

This project is financially supported by the Norwegian Research Council under the INCLINE project (274712).

## CONFLICT OF INTEREST STATEMENT

The authors declare no conflict of interest.

## Supporting information


Appendix S1.

Appendix S2.

## Data Availability

Data are available on the Open Science Framework (https://osf.io/h7qau/), (https://doi.org/10.17605/OSF.IO/H7QAU), along with the first stage of the registred report, and the accompanying script for data cleaning and analysis is available on Zenodo (https://doi.org/10.5281/zenodo.7589620).
