# Supplementary material for: A test of local adaptation to drought in germination and seedling traits in populations of two alpine forbs across a 2000 mm/year precipitation gradient
Source: Ecol Evol. 2023 Feb 7;13(2):e9772. doi: 10.1002/ece3.9772 (PMC9905427; doi:10.1002/ece3.9772)
Supplement: Supplementary file 1 — Appendix S1. [file ECE3-13-e9772-s001.docx]

Appendix 1: Synchrony
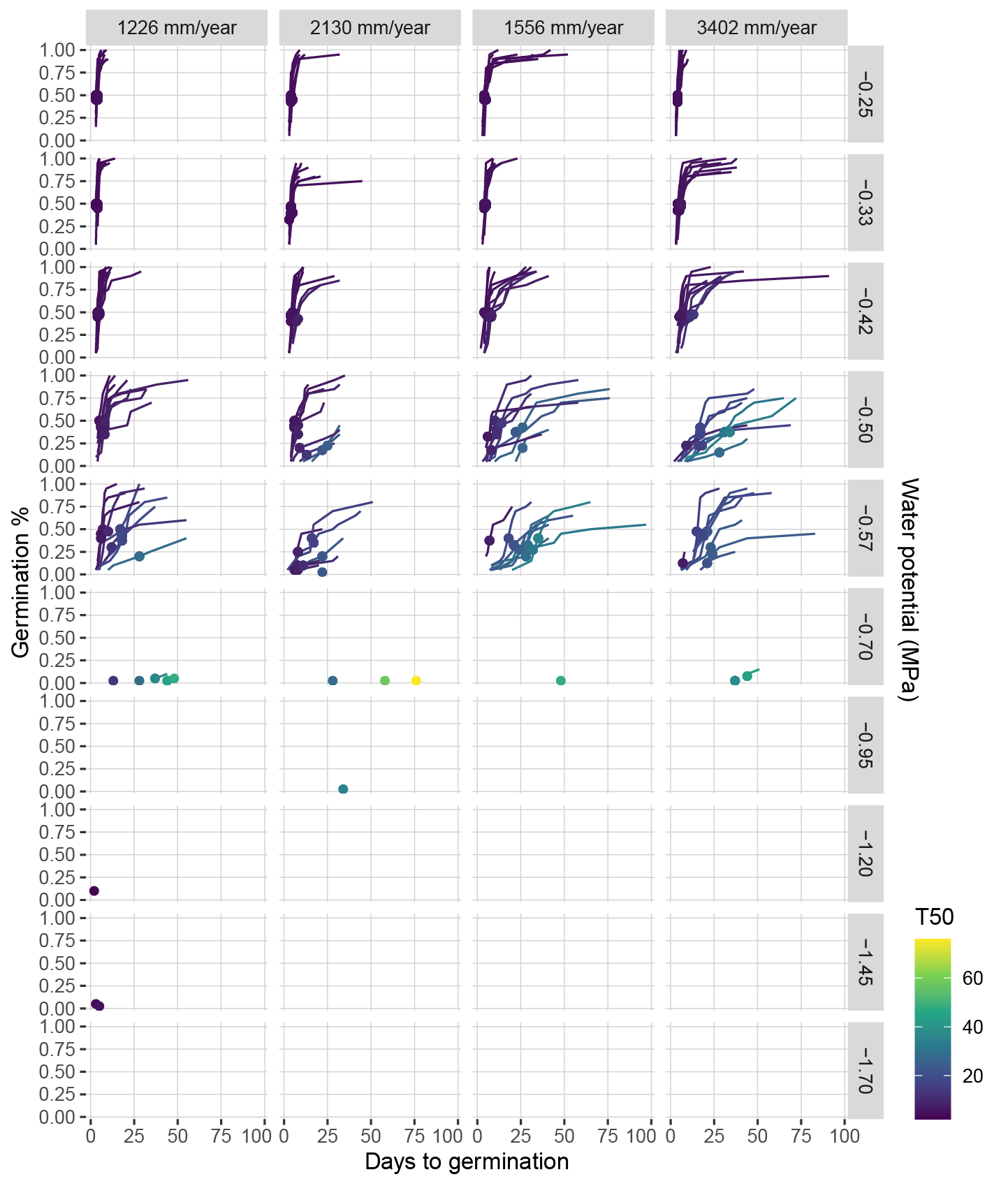

**Figure A1.1:** Accumulated germination percentage per Petri dish over time for *Veronica alpina* from four populations along a precipitation gradient (columns), and ten different water potential treatments (rows). Each line represents a replicate/Petri dish at that treatment, time to 50 % germination (T50) is represented with a point, and coloring from dark blue with the fastest germination rates, via green to yellow for the slowest germination rates.


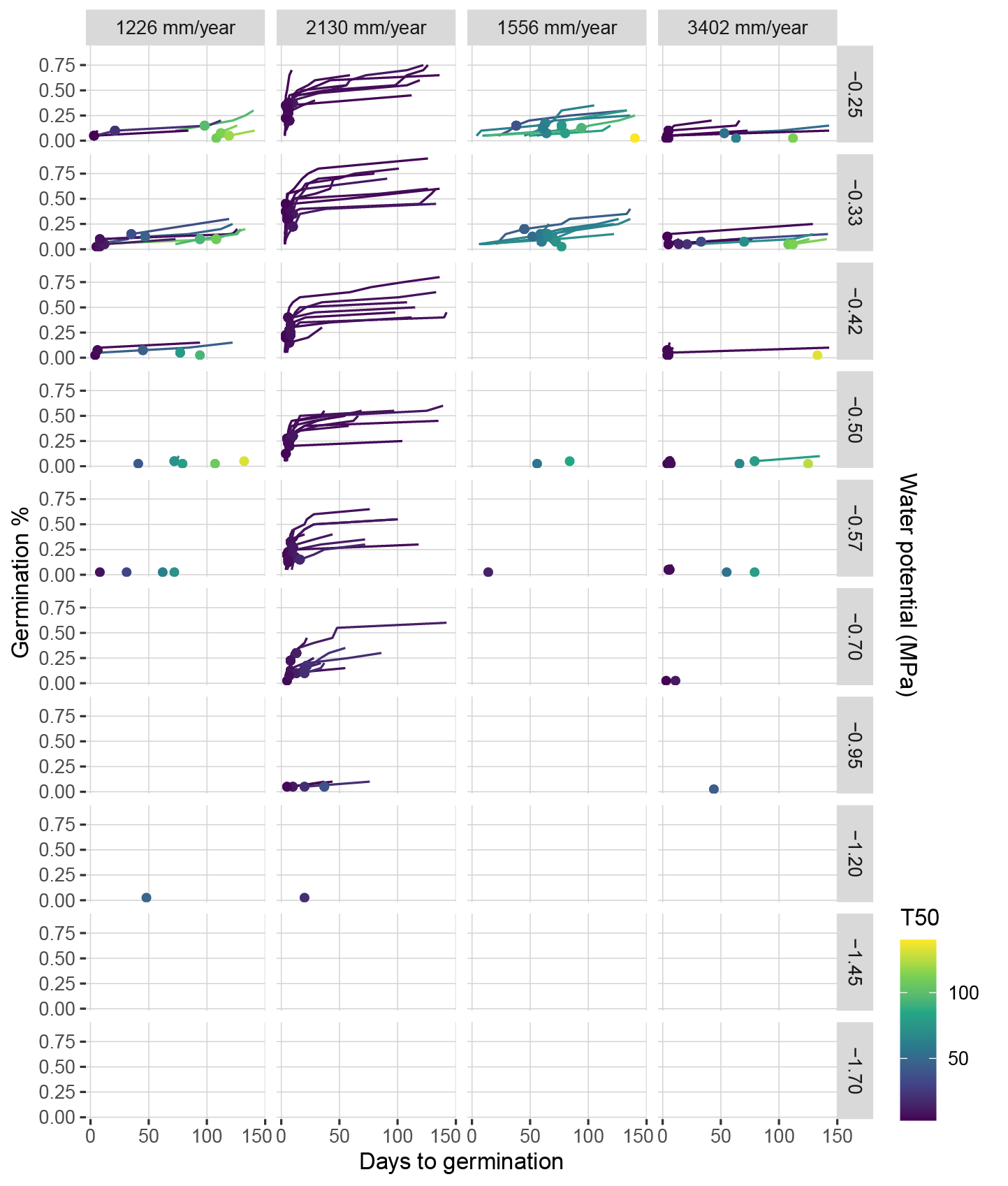


**Figure A1.1:** Accumulated germination percentage per Petri dish over time for *Sibbaldia procumbens* from four populations along a precipitation gradient (columns), and ten different water potential treatments (rows). Each line represents a replicate/Petri dish at that treatment, time to 50 % germination (T50) is represented with a point, and coloring from dark blue with the fastest germination rates, via green to yellow for the slowest germination rates.
