# Supplementary material for: A test of local adaptation to drought in germination and seedling traits in populations of two alpine forbs across a 2000 mm/year precipitation gradient
Source: Ecol Evol. 2023 Feb 7;13(2):e9772. doi: 10.1002/ece3.9772 (PMC9905427; doi:10.1002/ece3.9772)
Supplement: Supplementary file 2 — Appendix S2. [file ECE3-13-e9772-s002.docx]

# Appendix 2: Models of seed mass

Results from Baysian model including seed mass and water potential as explanatory variables for the different germination responses and seedling traits. Table A2.1 shows all the results, while Figure A2.1 and A2.2 shows time to max germination and above ground biomass for *Veronica alpina,* and A2.3 shows time to 50% germination for *Sibbaldia procumbens*. In addition to the germination percentage figure presented in the main paper, these relationships represented by figures are the only significant relationships between seed mass and germination responses and seedling traits either directly or indirectly through the interaction with water potential.

**Table A2.1:** Model output from Bayesian models on how water potential affects germination metrics and seedling traits from different populations with different seed mass. For each model, the water potential and seed mass of each observation was standardized by subtracting the mean water potential and dividing by the standard deviation. Each number is the model estimate based on one unit of the standardized water potential and indicates the effect of decreasing drought (going from low to high water potential). Bolded numbers in the median column are those where the 95% credible interval (CI) does not overlap zero, indicating the significance level with a threshold of 0.05 in a frequentist framework. The. The populations come from different precipitation levels, which does not significantly correlate with the seed mass of the populations. Seeds were sampled at alpine sites in Western Norway. Sample size of the germination traits are number of Petri dishes, while the germination traits use individual seedlings, hence the difference in sample size between models.

| ***Variable*** | ***Median*** | ***Std.dev*** | ***Lower 95% CI*** | ***Upper 95% CI*** | ***Median*** | ***Std.dev*** | ***Lower 95% CI*** | ***Upper 95% CI*** |
| --- | --- | --- | --- | --- | --- | --- | --- | --- |
|  | *Germination percentage (%)* | | | | | | | |
|  | *Veronica alpina* (n = 285) | | | | *Sibbaldia procumbens* (n = 271) | | | |
| Intercept | **-1.006** | 0.068 | -1.124 | -0.886 | **-3.496** | 0.198 | -3.831 | -3.110 |
| Seed mass | **-0.253** | 0.058 | -0.367 | -0.141 | **1.980** | 0.183 | 1.612 | 2.298 |
| Water potential | **4.794** | 0.173 | 4.515 | 5.062 | **2.483** | 0.213 | 2.058 | 2.837 |
| Seed mass:Water potential | 0.090 | 0.132 | -0.173 | 0.348 | **-0.927** | 0.193 | -1.251 | -0.532 |
| R^2^ | 0.463 |  | 0.455 | 0.478 | 0.625 |  | 0.609 | 0.643 |
|  | *Time to max germination (days)* | | | | | | | |
|  | *Veronica alpina* (n = 285) | | | | *Sibbaldia procumbens* (n = 271) | | | |
| Intercept | **3.138** | 0.045 | 3.050 | 3.227 | **4.402** | 0.069 | 4.272 | 4.540 |
| Seed mass | 0.011 | 0.044 | -0.075 | 0.099 | 0.004 | 0.068 | -0.129 | 0.136 |
| Water potential | **-0.459** | 0.046 | -0.550 | -0.368 | **0.212** | 0.072 | 0.068 | 0.352 |
| Seed mass:Water potential | **0.125** | 0.046 | 0.033 | 0.214 | -0.059 | 0.069 | -0.194 | 0.080 |
| R^2^ | 0.383 |  | 0.288 | 0.472 | 0.0491 |  | 0.00552 | 0.102 |
|  | *Time to 50% germination (days)* | | | | | | | |
|  | *Veronica alpina* (n = 285) | | | | *Sibbaldia procumbens* (n = 271) | | | |
| Intercept | **2.014** | 0.039 | 1.937 | 2.091 | **3.278** | 0.085 | 3.115 | 3.450 |
| Seed mass | -0.030 | 0.039 | -0.107 | 0.045 | **-0.832** | 0.098 | -1.027 | -0.642 |
| Water potential | **-0.622** | 0.039 | -0.699 | -0.544 | 0.006 | 0.086 | -0.164 | 0.171 |
| Seed mass:Water potential | -0.001 | 0.039 | -0.078 | 0.076 | -0.030 | 0.096 | -0.221 | 0.160 |
| R^2^ | 0.682 |  | 0.639 | 0.714 | 0.410 |  | 0.305 | 0.503 |
|  | *Belowground biomass (g)* | | | | | | | |
|  | *Veronica alpina* (n = 285) | | | | *Sibbaldia procumbens* (n = 271) | | | |
| Intercept | **-9.169** | 0.044 | -9.254 | -9.083 | **-9.752** | 0.049 | -9.847 | -9.655 |
| Seed mass | -0.024 | 0.043 | -0.109 | 0.061 | 0.051 | 0.052 | -0.051 | 0.155 |
| Water potential | **-0.492** | 0.043 | -0.575 | -0.408 | -0.054 | 0.050 | -0.152 | 0.044 |
| Seed mass:Water potential | -0.038 | 0.043 | -0.121 | 0.047 | -0.063 | 0.061 | -0.183 | 0.057 |
| Variance of traits | 1.052 | 0.015 | 1.024 | 1.083 | 1.119 | 0.031 | 1.061 | 1.182 |
| Variance random effect | 0.497 | 0.036 | 0.432 | 0.572 | 0.130 | 0.077 | 0.028 | 0.303 |
| R^2^ | 0.154 |  | 0.111 | 0.197 | 0.00991 |  | 0.000138 | 0.0252 |
|  | *Aboveground biomass (g)* | | | | | | | |
|  | *Veronica alpina* (n = 285) | | | | *Sibbaldia procumbens* (n = 271) | | | |
| Intercept | **-9.082** | 0.022 | -9.124 | -9.038 | **-8.111** | 0.031 | -8.170 | -8.050 |
| Seed mass | 0.000 | 0.022 | -0.042 | 0.043 | 0.036 | 0.032 | -0.026 | 0.098 |
| Water potential | **-0.191** | 0.022 | -0.233 | -0.148 | -0.007 | 0.031 | -0.066 | 0.054 |
| Seed mass:Water potential | **-0.049** | 0.022 | -0.092 | -0.006 | 0.023 | 0.037 | -0.049 | 0.094 |
| Variance of traits | 0.748 | 0.011 | 0.727 | 0.769 | 0.638 | 0.018 | 0.604 | 0.675 |
| Variance random effect | 0.211 | 0.021 | 0.172 | 0.254 | 0.143 | 0.044 | 0.049 | 0.223 |
| R^2^ | 0.0604 |  | 0.0376 | 0.0854 | 0.00899 |  | 0.0000690 | 0.0245 |
|  | *Root:shoot ratio (g/g)* | | | | | | | |
|  | *Veronica alpina* (n = 285) | | | | *Sibbaldia procumbens* (n = 271) | | | |
| Intercept | **-0.095** | 0.039 | -0.172 | -0.019 | **-1.653** | 0.045 | -1.740 | -1.565 |
| Seed mass | -0.022 | 0.039 | -0.098 | 0.054 | 0.015 | 0.045 | -0.075 | 0.104 |
| Water potential | **-0.302** | 0.039 | -0.378 | -0.226 | -0.024 | 0.045 | -0.114 | 0.065 |
| Seed mass:Water potential | 0.005 | 0.039 | -0.070 | 0.081 | -0.081 | 0.050 | -0.180 | 0.018 |
| Variance of traits | 1.029 | 0.015 | 1.001 | 1.058 | 1.034 | 0.029 | 0.979 | 1.093 |
| Variance random effect | 0.432 | 0.033 | 0.373 | 0.501 | 0.114 | 0.070 | 0.027 | 0.280 |
| R^2^ | 0.0710 |  | 0.0402 | 0.104 | 0.00877 |  | 0.0000808 | 0.0240 |


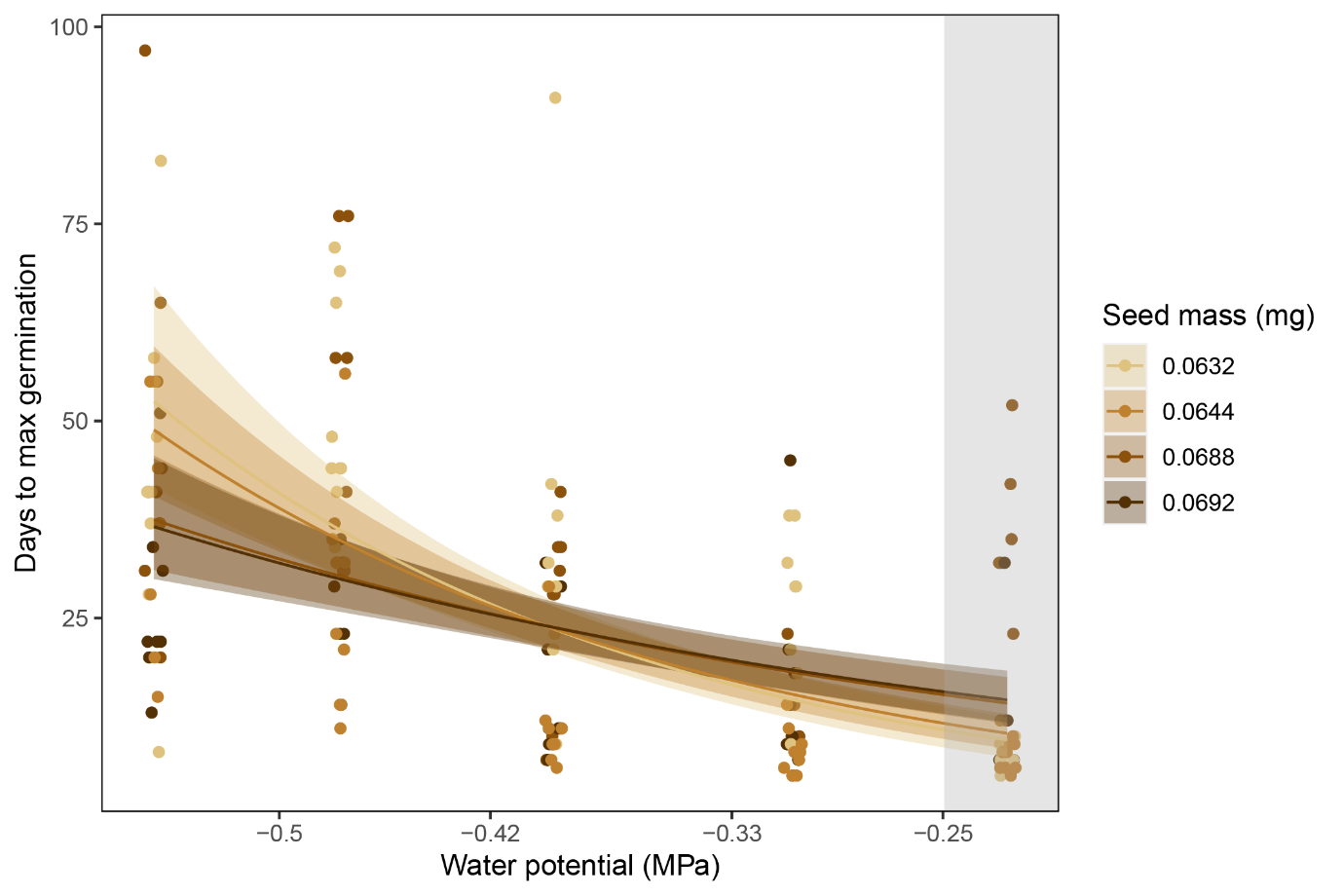
 **Figure A2.1:** Days to max germination for *Veronica alpina*, across different water potentials (MPa). -0.25 MPa represents pure agar medium with no drought treatment (marked with grey). The colors are based on the seed mass of the different populations within each species, with the dark brown represents the population with the heaviest seeds, and the beige the population with the lightest seeds (note the size difference between species). Seeds were sampled at four alpine sites in Western Norway, across a large precipitation gradient. Matching the seed mass to precipitation for *V. alpina* the population with 0.0632 mg seed mass comes from the population with an annual precipitation average between 2009-2019 of 3402 mm/year, 0.0644 mg from 1226 mm/year, 0.0688 mg from 2130 mm/year and 0.0692 mg from the population with 1561 mm/year. The lines represent predicted means with 95% credible interval envelopes.


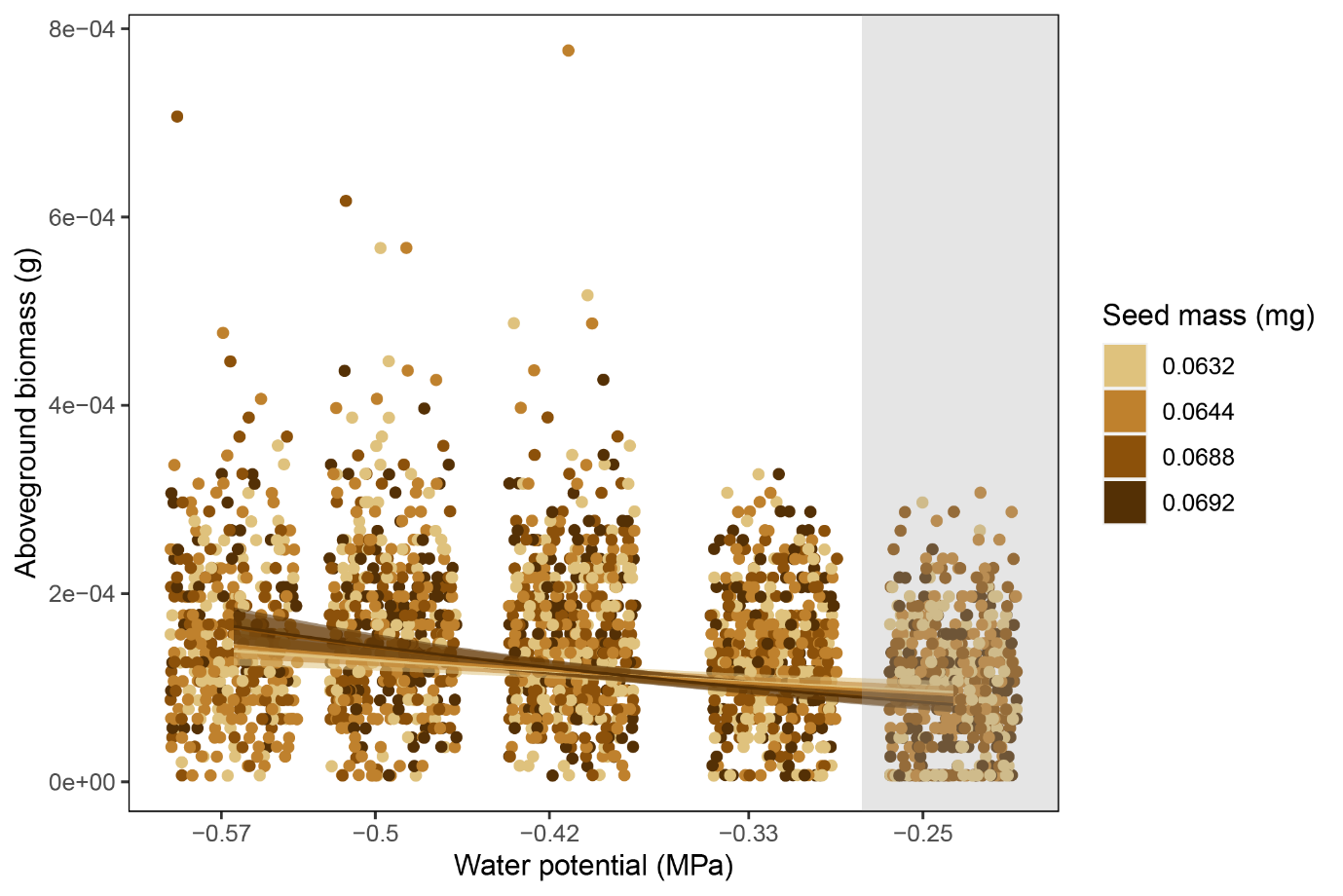


**Figure A2.2:** Aboveground biomass for seedlings of *Veronica alpina*, sampled one week after they developed their first pair of true leaves across different water potentials (MPa). -0.25 MPa represents pure agar medium with no drought treatment (marked with grey). The colors are based on the seed mass of the different populations within each species, with the dark brown represents the population with the heaviest seeds, and the beige the population with the lightest seeds (note the size difference between species). Seeds were sampled at four alpine sites in Western Norway, across a large precipitation gradient. Matching the seed mass to precipitation for *V. alpina* the population with 0.0632 mg seed mass comes from the population with an annual precipitation average between 2009-2019 of 3402 mm/year, 0.0644 mg from 1226 mm/year, 0.0688 mg from 2130 mm/year and 0.0692 mg from the population with 1561 mm/year. The lines represent predicted means with 95% credible interval envelopes.


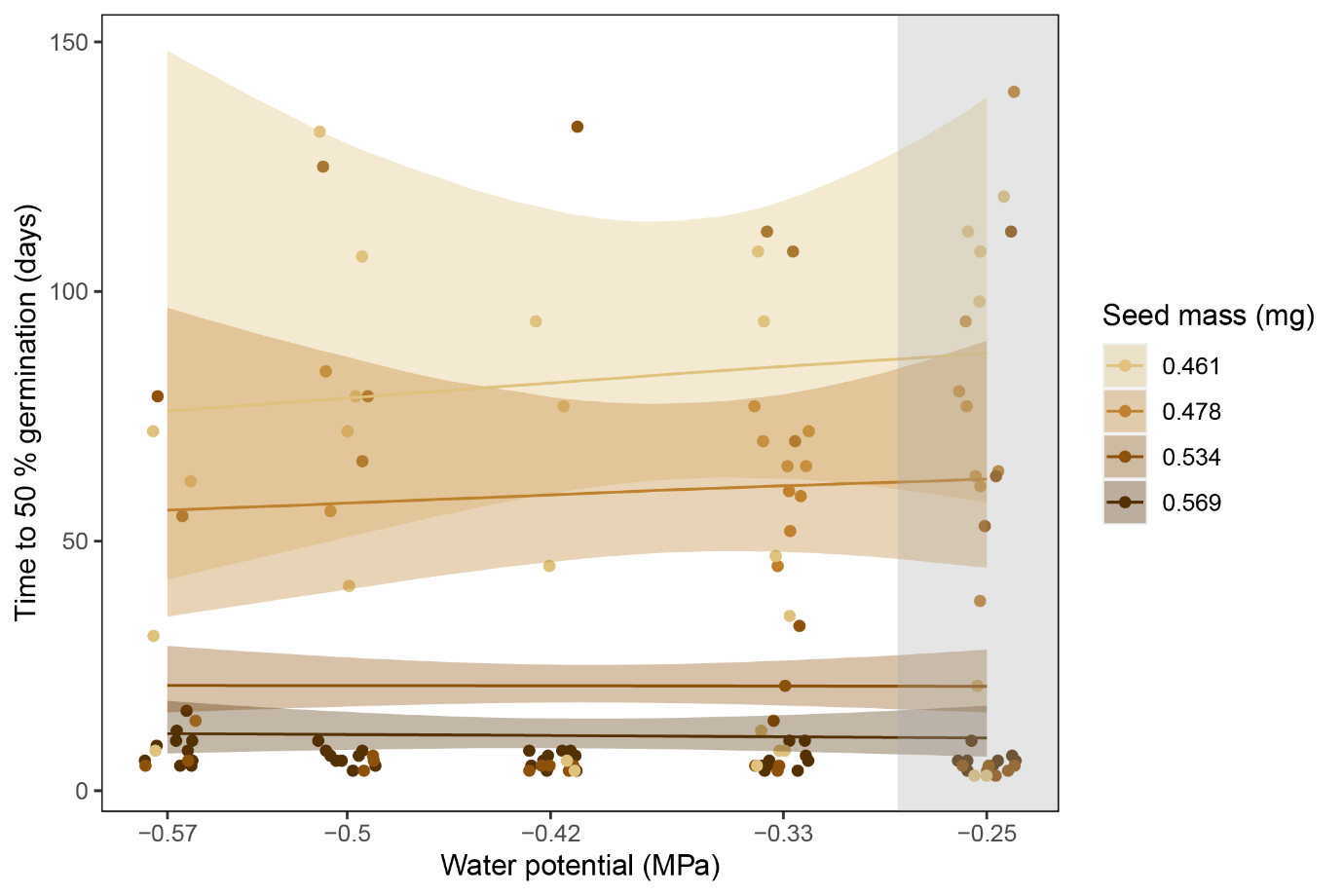


**Figure A2.3:** Time to 50% germination for *Sibbaldia procumbens*, across different water potentials (MPa). -0.25 MPa represents pure agar medium with no drought treatment (marked with grey). The colors are based on the seed mass of the different populations within each species, with the dark brown represents the population with the heaviest seeds, and the beige the population with the lightest seeds (note the size difference between species). Seeds were sampled at four alpine sites in Western Norway, across a large precipitation gradient. Matching the seed mass to precipitation for *S. procumbens* the population with seeds of 0.461 mg comes from the population with an average from 2009-2019 in annual precipitation of 1226 mm/year, 0.478 mg from 2130 mm/year, 0.534 mg from 3402 mm/year and 0.569 mg from 1561 mm/year. The lines represent predicted means with 95% credible interval envelopes.
